# Foraging and mating behaviors of *Hypsignathus monstrosus* at the bat‐human interface in a central African rainforest

**DOI:** 10.1002/ece3.10240

**Published:** 2023-07-08

**Authors:** Elodie Schloesing, Alexandre Caron, Rémi Chambon, Nicolas Courbin, Morgane Labadie, Roch Nina, Frida Mouiti Mbadinga, Wilfrid Ngoubili, Danficy Sandiala, N’Kaya Tobi, Mathieu Bourgarel, Hélène M. De Nys, Julien Cappelle

**Affiliations:** ^1^ Faculté des Sciences Université de Montpellier Montpellier France; ^2^ CIRAD, BIOS, UMR ASTRE Montpellier France; ^3^ Faculté des Sciences et Techniques Université Marien Ngouabi Brazzaville Democratic Republic of the Congo; ^4^ Ministère de l'Agriculture, de l'Elevage et de la Pêche Direction Générale de l'Elevage Brazzaville Democratic Republic of the Congo; ^5^ Ministère de l'Economie Forestière Direction de la Faune et des aires Protégées Brazzaville Democratic Republic of the Congo; ^6^ Faculdade de Veterinaria Universidade Eduardo Mondlane Maputo Mozambique; ^7^ Université de Rennes 1, unité BOREA MNHN, CNRS 8067, SU, IRD 207, UCN UA Rennes France; ^8^ Centre d'Ecologie Fonctionnelle et Evolutive, UMR 5175 Université de Montpellier, CNRS, EPHE, IRD Montpellier France; ^9^ CIRAD, BIOS, UMR ASTRE Harare Zimbabwe

**Keywords:** Ebola virus, GPS telemetry, hammer‐headed bat, movement ecology, republic of Congo, resource selection function

## Abstract

Studying wildlife space use in human‐modified environments contributes to characterize wildlife‐human interactions to assess potential risks of zoonotic‐pathogens transmission, and to pinpoint conservation issues. In central African rainforests with human dwelling and activities, we conducted a telemetry study on a group of males of *Hypsignathus monstrosus,* a lek‐mating fruit bat identified as a potential maintenance host for Ebola virus. During a lekking season in 2020, we investigated the foraging‐habitat selection and the individual nighttime space use during both mating and foraging activities close to villages and their surrounding agricultural landscape. At night, marked individuals strongly selected agricultural lands and more generally areas near watercourses to forage, where they spent more time compared to forest ones. Furthermore, the probability and duration of the presence of bats in the lek during nighttime decreased with the distance to their roost site but remained relatively high within a 10 km radius. Individuals adjusted foraging behaviors according to mating activity by reducing both the overall time spent in foraging areas and the number of forest areas used to forage when they spent more time in the lek. Finally, the probability of a bat revisiting a foraging area in the following 48 hours increased with the previous time spent in that foraging area. These behaviors occurring close to or in human‐modified habitats can trigger direct and indirect bat‐human contacts, which could thus facilitate pathogen transmission such as Ebola virus.

## Graphical abstract

Wild animals may adapt or not to human‐altered landscapes and interact with humans. In this field study, we highlighted that an African fruit bat species primarily forages in agricultural lands and watercourses that surround small forestry villages. We also provide insights about space use dynamic of individuals during their nocturnal foraging and breeding activities. These data improve basic knowledge about the interface between fruit bats and human rural communities and highlight potential mechanisms for pathogen transmission.

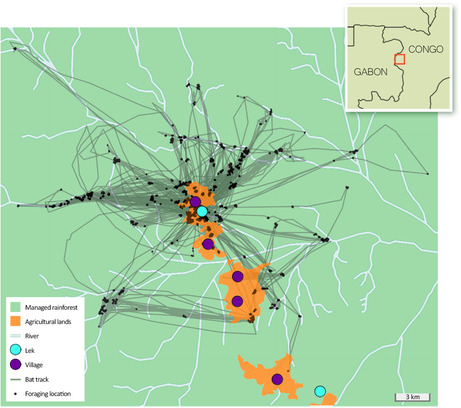

## 1 INTRODUCTION

Foraging and breeding activities are essential for organisms (Alcock, 2013; Schoener, 1971), and animals have to adapt their movements to optimize their fitness from each behavior (Reaney, 2007), resulting in space use patterns. In the human‐modified landscapes that today dominate a large proportion of terrestrial ecosystems, space use patterns of individuals may lead to contact with domesticated animals and humans. These contacts can result in threats to wild animal populations when they are unsustainably used and trigger intra‐ and inter‐specific pathogen transmission (e.g., through contaminated leftover food or close contacts between individuals; Boulinier et al., 2016; Uchii et al., 2011). Habitat selection patterns of wild species for their foraging and breeding activities are therefore particularly relevant to identify ecological drivers partly leading to pathogen transmission (e.g., environments where risks of human exposure to infectious agents are the highest; Dougherty et al., 2018). A more comprehensive study of human‐wildlife interactions should consider the dynamics of daily activities of animal species (i.e., the duration and frequency of visits within the foraging and breeding areas), and how they balance their investment between foraging and reproductive behaviors. Yet, such complementary analyses have rarely been assessed, preventing a better understanding of animal space use patterns (Martin et al., 2009). Rigorous identification of the drivers of movements associated with foraging and reproductive behaviors would then allow to design efficient management strategies benefiting both public health and species conservation.

Some animal orders have received considerable attention given their taxonomic diversity and higher propensity to be sources of zoonotic infections (e.g., rodents, primates, bats; Mollentze & Streicker, 2020). The orders comprising host species thriving in anthropogenic habitats are of major concern since they are more likely to be in contact with humans (Nading, 2013). Among them, fruit bats are important hosts of emerging viruses (Calisher et al., 2006), some of which were involved in severe and recent outbreaks in human populations (Cappelle et al., 2020; Sharma et al., 2019). Fruit bat species display highly diversified daily foraging activity patterns (e.g., visiting one or several foraging areas with varying duration and re‐visitation rates; McEvoy et al., 2021; Schloesing et al., 2020) and mating systems (mainly polygamous using a central place such as harems and leks; Crichton & Krutzsch, 2000). These movements within human‐modified ecosystems must be better understood from an ecological and epidemiological perspective. Despite the fact that several studies on the movement patterns of fruit bats in anthropogenic landscapes have been conducted (for a review, see Williams‐Guillén et al., 2016), habitat selection, as well as frequency and duration of local movements during breeding and foraging activities remain poorly documented.

The present study focuses on foraging‐habitat selection, and both foraging and breeding activity patterns at the individual level in the hammer‐headed bat (*Hypsignathus monstrosus*). Although listed as “least concern” on the IUCN red list, the species is hunted for bushmeat (Mildenstein et al., 2016) and the IUCN reported a continuing decline of mature individuals (Tanshi, 2016). This is one of the eight fruit bats species suspected to be involved in the circulation and potential maintenance of Ebola virus (De Nys et al., 2018). *H. monstrosus* would belong to bush‐meat species involved in direct transmission pathways of Ebola virus to humans (Leroy et al., 2009), but indirect ones remaining poorly understood despite their relevance (e.g., contacts after the contamination of food items; Baudel et al., 2019). Although individuals of *H. monstrosus* have been caught in a wide range of habitats, from primary forest to urban areas, during several inventory surveys (Niamien et al., 2010; Waghiiwimbom et al., 2020), habitat‐type preference at population scale and daily foraging pattern of individuals are unknown. Furthermore, the species displays a lek mating behavior during biannual breeding periods (Bradbury, 1977). A lek is a local aggregation (in a fixed site without food resources) of numerous small male territories used to attract females at night. Such an aggregation is likely to increase contact rates among conspecifics and may enhance pathogen transmission within the host population (Benavides et al., 2012; Bradbury, 1977), as well as direct and indirect contacts with humans when leks are established close to human settlements.

During one breeding period in 2020, we collected GPS data from 28 individuals using a lek site located in the vicinity of a village in the Republic of Congo, in an area where Ebola outbreaks were identified from 2003 to 2005 (Rugarabamu et al., 2020). The proximity of the lek to the village allowed us to study a potential bat‐human interface. The objectives were (i) to characterize the overall foraging‐habitat selection of males using resource selection function; then to investigate the individual patterns related to the nighttime use of (ii) the lek (i.e., visit probability and duration) and (iii) foraging areas (i.e., location of areas in relation to the lek, number of foraging areas, visit duration, and revisitation probability over consecutive nights). Finally, we combined these results to highlight movement patterns resulting from trade‐offs between both activities and how human‐induced changes in rainforests could influence the interaction of bats with humans, including pathogen‐transmission risks.

## 2 METHODS

### 2.1 Study region

**FIGURE 1:**
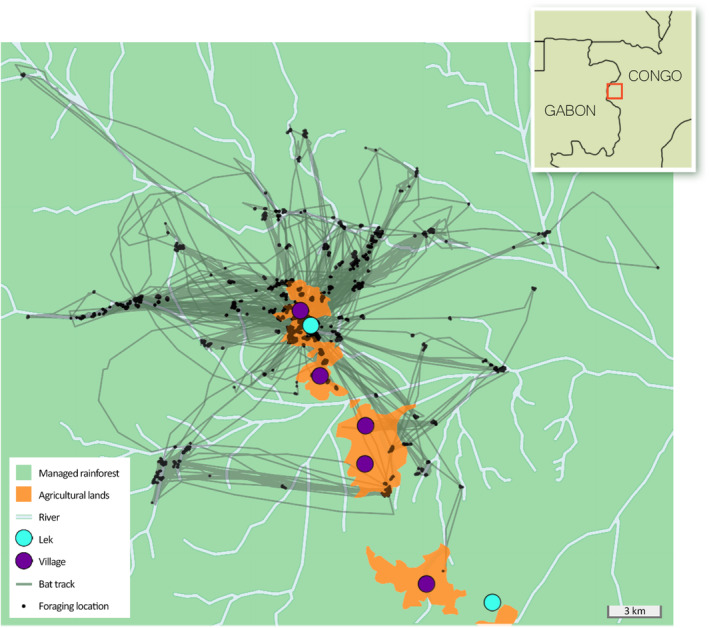
Study site composition, global positioning system (GPS) tracks, and foraging locations of the 28 males of *Hypsignathus monstrosus* studied. The northern lek site is the place used by these individuals to mate.

The study was conducted in a western region of the Republic of Congo (Kellé district, “Western Cuvette” department) from January to February 2020, during one of two biannual breeding periods (December–February and June–August). This area hosts a population of *H*. monstrosus, in which males use a lek site (14.181°E, 0.204°N) located 800 m from the “Ndjoukou” village, in a primary forest along a stream (Likouala river; Figure 1). The lek gathers male display territories (typically at least separated by 10 m; Bradbury, 1977) over a total surface of 25 ha. We estimated this surface by mapping calling bats during field surveys at night. The landscape within a 25 km radius from the lek site (i.e., according to the furthest distance reached by a bat during the study period, a posteriori determined by GPS data) is almost entirely covered by a rainforest managed since 2008 by Congo Deija Wood Industries that includes primary and secondary forest patches. Otherwise, the landscape includes agricultural lands in which six small villages are nested (including “Ndjoukou” village). Overall, agricultural lands correspond to patchworks of fields (17%; manly manioc *Manihot esculenta*) and secondary vegetation successions (83%; mainly forest of pioneer trees that regrow following the cessation of agricultural activities). In addition, many cultivated trees, identified during field surveys, are dispersed near villages and fields: bananas and plantains (*Musa* spp.), papayas (*Carica papaya*), ananas (*Ananas comosus*), avocado (*Persea Americana*), safou trees (*Dacryodes edulis*), oil palm (*Elaeis guineensis*), citruses (*Citrus* spp.), and cacao trees (*Theobroma cacao*).

To perform the spatial analyses presented in the article, we mapped the study region by identifying the limits of the managed rainforest and agricultural lands. We used a satellite imagery (sentinel‐2; spatial resolution of 10 m‐pixel size acquired on February 15 and August 08, 2020), and a map of normalized difference vegetation Index (NDVI; spatial resolution of 30 m‐pixel size derived from a Landsat‐8 image acquired on January 28, 2020) to identify the vegetation dynamics characterizing agricultural lands (Bellón et al., 2017). We mapped the river system by combining satellite imagery (sentinel‐2) and elevation mapping (spatial resolution of 30 m‐pixel size from ASTER Global Digital Elevation Model V003).

Some fruiting trees consumed by bats are especially abundant along rivers in central Africa (e.g., *Ficus* spp.; Gautier‐Hion & Michaloud, 1989). To improve our knowledge about the distribution of such specific resources along rivers in the study region, we monitored the distribution of fruiting *Ficus mucuso* along a 40 km‐long road crossing the region (both the managed rainforest and agricultural lands) with a variable distance to rivers (see Appendix S1).

### 2.2 GPS‐data collection

Individuals were caught during the nights from 9th to 14th January 2020, using canopy mist nets directly deployed within the lek site or on a path leading to that site. Each logger (model: bird solar 15 g, e‐obs Digital Telemetry) was previously fixed on a homemade collar “cape” (for the design, see Olson et al., 2019). The collar was sutured around the bat's neck using catgut suture (USP size 3–0) and surgical knots that were presumed to last for at least 1 month. As telemetry device should aim for 5% (or less) of the bat weight (O'Mara et al., 2014), only adult males were equipped due to their larger weight in comparison to females and juveniles. These individuals were anesthetized by an injection of Medetomidine into the pectoral muscle (Epstein et al., 2011). The total weight of a collar (16.3 g) represented on average 4.1% ± 0.5 SD (range: 3.6–5.7%) of individual body mass (mean ± SD: 401.0 g ± 42.9, range: 287–455). Bats were woken up with an intramuscularly injection of Atipamezol and were kept in separate cages during recovery from anesthesia (Epstein et al., 2011). Bats were handled in accordance with guidelines approved by the American Society of Mammologists (Sikes & Gannon, 2011; Sikes and the Animal Care and Use Committee of the American Society of Mammalogists, 2016). Sugar water was offered to individuals and they were finally released during the few hours following capture.

The loggers were turned on in the evening following the capture event to avoid potential behavioral biases related to capture. Loggers collected GPS locations and 3D accelerometer data. Data were downloaded with a maximum transfer distance of 10 km from four permanent base stations connected to antennas. A total of 32 bats were equipped with loggers but three were discarded for this study because of the early loss of the collar and one because of insufficient data. The 28 loggers with two different settings collected GPS locations and accelerometer data (16.7 Hz burst during 15 s every 2 min; Grundy et al., 2009) from 10th to 30th January. A first group (“group 1”) of 16 individuals was monitored from 17:45 to 06:00 (UTC + 01:00) during a mean of 9.8 nights ±2.0 SD (range: 5–12) with one location recorded every 5 min. A second group (“group 2”) of 12 individuals was monitored from 18:05 to 06:00 during a mean of 14.2 nights ±7.0 (range: 3–21). One location was recorded every 5 min during high‐activity bouts (i.e., flying; accelerometer variance threshold >10,000; Brown et al., 2012), and one location every 30 min otherwise (mainly resting and foraging). The GPS settings of the second group allowed us to extend battery life for a part of bats equipped, promoting the identification of areas visited by them across an extended period.

The study was approved by the local authority responsible for wildlife research: the Ministry of Agriculture, Livestock and Fisheries and the French VetAgro Sup ethic committee approved the study (number 1805‐V2, July 3, 2018) as there was no Animal ethics committee at that time on the Republic of Congo.

### 2.3 Behavioral state identification

We considered three typical movement types for bats related to their foraging and mating activities: stationary bout (fruit consumption, display calls, and resting), short flight (between close food patches within a foraging area or between display territories), and commuting flight (long distance and directional movements between different foraging areas or between a foraging area and the lek site). We used a hidden Markov model (HMM) based on smoothed speed and absolute angle values calculated between two successive locations (McClintock & Michelot, 2018; Patin et al., 2020) to attribute a unique behavioral state (i.e., movement type) to each location for each bat from group 1 (regular and high acquisition rate being required). We assumed a gamma distribution for speed and a von Mises distribution for angle. We fitted HMM with the “momentuHMM” R package (McClintock & Michelot, 2018).

Regarding group 1, the mean values of smoothed speed resulting from the modeling procedure were (i) 2.4 m/min ± 1.2 SD (range: 0.1–6.7) for stationary bouts, (ii) 9.1 ± 5.1 (range: 0.6–33.5) for short flights, and (iii) 134.3 ± 141.4 (range: 0.4–796.2) for commuting flights. These values were consistent with the current knowledge on the species (Carpenter, 1986). Behavioral state identification was then generalized to locations from group 2. For this purpose, smoothed speed was computed for all these locations and the maximal value of smoothed speed obtained for short flights in group 1 (33.5 m/min) was used as a threshold. The second location of each pair of consecutive locations, for which the speed calculated was above this threshold, was associated with commuting flights and discarded since the HMM approach could not be directly carried out for group 2. This method maximized the sample size of the short flights and stationary bouts. If applied on group 1, the locations retained (short flights and stationary bouts) increased by only 3.5% compared to the segmentation (HMM) method, indicating a good correlation between methods.

For both groups, short flight and stationary bout locations within the lek site area were considered as related to the mating activity and related to the foraging activity otherwise. Finally, stationary locations recorded at the very beginning or ending of the night were associated with the bat's daily resting (an individual typically roosts alone or within a small group in a given place for a few days, before moving to another place located a few meters or kilometers away; Bradbury, 1977) and were consequently excluded from the datasets analyzed.

All individuals were included in the analyses related to the foraging‐habitat selection and the probability of a bat visiting the lek site, whereas the rest of analyses (requiring higher temporal resolution) were limited to group 1.

### 2.4 Foraging area characterization

Following Schloesing et al. (2020), we characterized the foraging areas used by each monitored bat (FAs; zones including one or several food patches where individuals actively search food, consume fruits, or rest), based on the concept of “area‐restricted search” (ARS; Kareiva & Odell, 1987). For a given bat, an ARS behavior includes a varying sequence of short flights and stationary bout locations (or a unique location when no sequence occurred) recorded outside the lek site and separated by at least one commuting flight. Each ARS behavior was performed in one FA that was delimited by creating the minimum convex polygon from all constitutive short flights and stationary bout locations, for each bat. Given that individuals commonly revisited FAs, overlapping ones were considered as a unique FA for each bat (following Schloesing et al., 2020). The corresponding habitat type (i.e., managed rainforest or agricultural lands) was attributed to each FA. We calculated the Geodesic distance (meters) between each FA and the lek site by averaging the distance of all constitutive locations to the centroid of the lek. Similarly, the closest distance to the river was calculated for each FA. The duration spent within each FA during a given night was calculated by cumulating the time elapsed between consecutive constitutive locations (if several visits of the same FA occurred during a night for a given bat, the duration was cumulated). Then, we determined FAs that were revisited on the following nights.

Appendix S2 presents data related to the field prospection of randomly selected foraging patches to describe potential food‐resource availability for bats.

### 2.5 Foraging‐habitat selection modeling

A resource selection function (RSF) was used to estimate foraging‐habitat selection (Johnson et al., 2013; Muff et al., 2020). RSF was based on a comparison between the environmental characteristics observed at the foraging GPS locations and those observed at random locations within the study area. The random locations were generated from the space available and accessible for the individuals (10 random locations for each GPS location) defined as the 95% utilization distribution (UD) of all GPS locations using a biased random bridge approach (Benhamou, 2011). This method consists of a kernel density estimation taking into account individual movements and provides parameters to consider GPS locations recorded at irregular time intervals (Benhamou, 2011; Dürr & Ward, 2014). As the lek acts as a spatial anchor resulting in a central‐place forager behavior for bats during the breeding season, we generated random locations considering a bivariate exponential distribution centered on the lek and of radius equal to the farthest boundary of the UD (*r* = 21.8 km; Monsarrat et al., 2013).

The RSF was fitted using a generalized linear mixed model approach (GLMMs with a binomial distribution for error and a logit link function; R software) to assess the relative probability of selection. We tested the effect of the habitat type (i.e., agricultural lands and managed rainforest), as a proxy of differences in fruit availability (i.e., diversity and abundance) and other factors (e.g., differences in human disturbance and predation risk). The effect of the closest distance to the river was also tested since some bat species are known to use rivers as landmarks for navigation (Furmankiewicz & Kucharska, 2009; Rydell et al., 2014), and since fruiting trees consumed by bats are particularly abundant along rivers in central Africa (e.g., *Ficus* spp.; Gautier‐Hion & Michaloud, 1989). A null model (intercept‐only) and four models including either the simple, additive, or interaction effect of these variables were computed. The interaction term was considered since the influence of the distance to the river on vegetation may vary with the habitat type (Fernandez‐Gimenez & Allen‐Diaz, 2001). Following Muff et al. (2020), individual‐specific random intercepts were fixed with a large variance, and random slopes according to individuals were used for the closest distance to the river (not for the habitat due to convergence issue).

A model was considered more competitive when its Akaike's information criterion (AICc; corrected for small sample sizes) was at least 2 units lower (ΔAICc) than others (in case of ambiguity, AICc weight was used—ωAICc). As a last step, the robustness of the selected model (i.e., predictive performance) was evaluated using k‐fold cross‐validation (Boyce et al., 2002).

### 2.6 Activity pattern modeling

#### 2.6.1 Mating activity

Given the potential importance of the lek regarding disease spread, we estimated the probability of a male bat visiting the lek and the time spent therein (when visited) during the night. More precisely, we tested the effect of distance between the lek and roost sites used by individuals on these variables to assess the adjustment of mating behaviors in relation to energy costs associated with distant flights and to identify a perimeter around the lek in which individuals are particularly connected due to their use of the lek. For this purpose, we identified the location of the roosts used by individuals on the basis of the GPS location preceding each nocturnal track (the location of the roost of individual was relatively stable for a few days for each bat, as already mentioned; Bradbury, 1977). Then, one GLMM was computed for each of the two responses (visitation probability: Binomial error distribution and logit link function; duration: Gamma error distribution and log link function), and the significance of the fixed effect was tested (significance level: *α* = 0.05).

#### 2.6.2 Foraging activity

Breeders are expected to adjust their nightly activity in response to the energy costs related to foraging (mainly travels) and mating behaviors (Shaffer et al., 2003). Activity patterns may specifically vary according to the habitat (vegetation type and distance to the river) of the sites used to forage (notably due to global food‐resource variation). Thus, we investigated whether the distance from a selected FA to the lek was influenced by the habitat type of the FA and the duration spent in the lek by a bat during the night, by comparing several GLMMs (a null model and models including all effect combinations; Gamma error distribution, log or identity link function when convergence issues occurred). Then, we tested whether the total number of FAs visited by a bat during the night in each habitat type was different, and whether the duration spent in the lek during the night differentially influenced this number for each habitat, by comparing several GLMMs (a null model, and models including only the simple effect of the habitat or also the interaction term between habitat and lek‐visitation duration; Poisson error distribution, log link function). In addition, we tested whether the total duration spent in a FA by a bat during the night was influenced by the habitat type of the FA, its distance to the river, and the duration spent in the lek during the night, by comparing several GLMMs (a null model and models including all effect combinations to a limit of one interaction per model without interaction term between lek‐visitation duration and FA‐river distance; Gamma error distribution, log link function). Since Ebola virus persists <72 h in a tropical environment (Nikiforuk et al., 2017), we investigated the probability of a bat revisiting a FA at least once in the following 48 h (two nights), in a context of potential local accumulation of pathogens and transmission risks. More specifically, we tested the effect of the habitat type of the FA, its distance to the river, and the duration spent in the FA during the night (a proxy of food‐resource quality of that specific site) by comparing several GLMMs (a null model and all possible models to a limit of one interaction per model, without interaction term between FA‐visitation duration and FA‐river distance; Binomial error distribution, logit link function).

Details about locations recorded and nighttime activities for each collared individual are presented in Appendix S4. All quantitative predictors were centered and scaled. Individual‐specific intercepts were considered in all models as random effect, as well as individual‐specific slopes when allowed. The model‐selection procedure was the same as for the RSF. In addition, marginal ($R_{m}^{2}$) and conditional ($R_{c}^{2}$) pseudo *R*‐squared were computed for models retained. The analyses were performed using R software. Details related to model construction (including random effects) and selection (including results and parameter estimates) are shown in Appendix S5 and Table 1.

**TABLE 1:** Estimates with their 95% confidence interval (CI) resulting from the model retained for foraging‐habitat selection pattern of males, and for mating and foraging activity patterns during the night, with pseudo‐*R* ^2^ (marginal and conditional) associated with each model.

Pattern	Response variable	Parameter	Mean estimate	CI (95%)	$R_{m}^{2}$	$R_{c}^{2}$	Figure
RSF	Probability of use	Location‐river distance	−1.27E^−3^	−1.82E^−3^; −7.12E^−4^	–	–	Figure 2
		Habitat	2.69	2.64; 2.73			
		Location‐river distance:Habitat	4.51E^−4^	1.15E^−3^; 1.95E^−3^			
Mating activity	Lek‐visitation probability	Intercept	−0.83	−2.50; 0.84	0.21	0.86	Figure 3a
		Roost‐lek distance	−2.55	−4.45; −0.65			
	Lek duration	Intercept	5.17	4.79; 5.54	0.39	0.55	Figure 3b
		Roost‐lek distance	−0.43	−0.61; −0.25			
Foraging activity	FA‐lek distance	Intercept	8.70	8.50; 8.89	0.63	0.69	Figure 4a
		Habitat	−1.51	−1.62; −1.41			
		Lek duration	0.13	0.01; 0.24			
		Habitati:Lek duration	−0.23	−0.35; −0.12			
	Number of FA	Intercept	0.53	0.27; 0.78	0.14	0.37	Figure 4b
		Habitat	−0.37	−0.55; −0.19			
		HabitatFor:Lek duration	−0.42	−0.65; −0.20			
		HabitatAgri:Lek duration	0.03	−0.18; 0.25			
	FA duration	Intercept	4.70	4.44; 4.96	0.20	0.35	Figure 4c
		Habitat	0.91	0.71; 1.11			
		FA‐river distance	0.13	0.02; 0.24			
		Lek duration	−0.16	−0.27; 0.05			
		Habitat: FA‐river distance	−0.33	−0.56; 0.09			
	FA‐revisitation probability	Intercept	2.26	1.57; 2.96	0.43	0.48	Figure 4d
		FA duration	2.32	1.45; 3.18			

*Note*: Habitat: parameter associated with the type “agricultural lands” (“the managed rainforest” being the implicit reference level in the intercept component). Location‐river distance: the closest distance between a given location and the river. Roost‐lek distance: the distance between the roost used by a bat during the night and the lek. Lek duration: the total duration spent in the lek by a bat during the night. FA‐river distance: the closest distance between a given foraging area (FA) and the river. FA duration: the total duration spent in a given FA by a bat during the night. See Appendix S5: Table S5 for more details about models. Values of intercept were not reported for the RSF pattern (fixed intercept; see the main text). Estimated marginal means were used to provide the adjusted estimates presented in the table and to obtain effect plots showing adjusted predictions for each model retained (“ggeffect” R package). The “Figure” column refers to the figure (effect plot) number in the main text.

## 3 RESULTS

### 3.1 Foraging‐habitat selection

**FIGURE 2:**
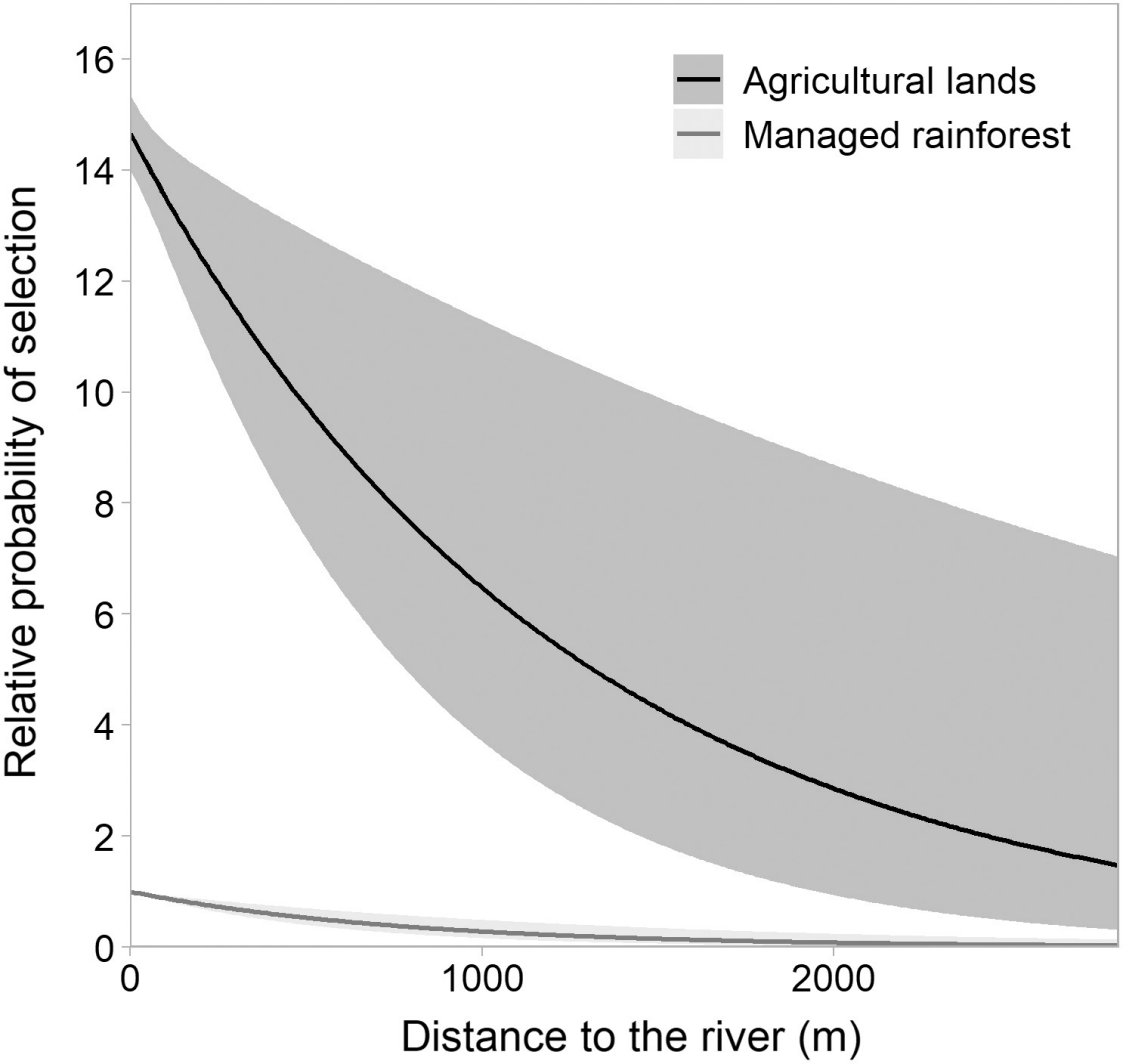
Estimation (with 95% CI; *n* = 28 individuals) of the relative probability of foraging‐habitat selection for males, in relation to the habitat type and the distance to the river (in m). The maximal distance to the river that could be reached by bats in agricultural lands was 2810 m and 5360 m in the managed rainforest (values above 2810 m were not represented in the figure for convenience).

The foraging‐habitat selection was influenced by the distance to the river, the habitat type, and their interaction (ΔAICc of the following model = 398.72; Figure 2). Bats have a lower relative probability to select a location far from the river for both habitats. However, the associated decreasing rate was higher for agricultural lands. Overall, bats were more likely to forage in agricultural lands than in the managed rainforest along the gradient of distance to the river. This difference was especially strong when bats foraged close to the river. The RSF model was moderately robust to cross‐validation (*r*s > 0.56; Appendix S5).

### 3.2 Mating activity

**FIGURE 3:**
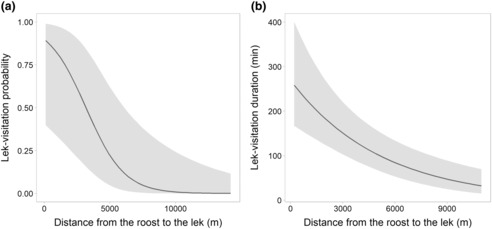
Estimation (with 95% CI) of (a) the probability of a bat visiting the lek site during the night (*n* = 28 individuals), in relation to the distance between the roost of the bat and the lek site (in m) and (b) the total duration spent in the lek during the night by a bat (in min), when visited (*n* = 11 individuals), in relation to the distance between the roost of the bat and the lek site (in m).

Bats roosting close to the lek were more likely to visit the lek (χ^2^ = 7.0, df = 1, *p* < .01; Figure 3a) and spent time therein (χ^2^ = 22.2, df = 1, *p* < .001; Figure 3b).

### 3.3 Foraging activity

**FIGURE 4:**
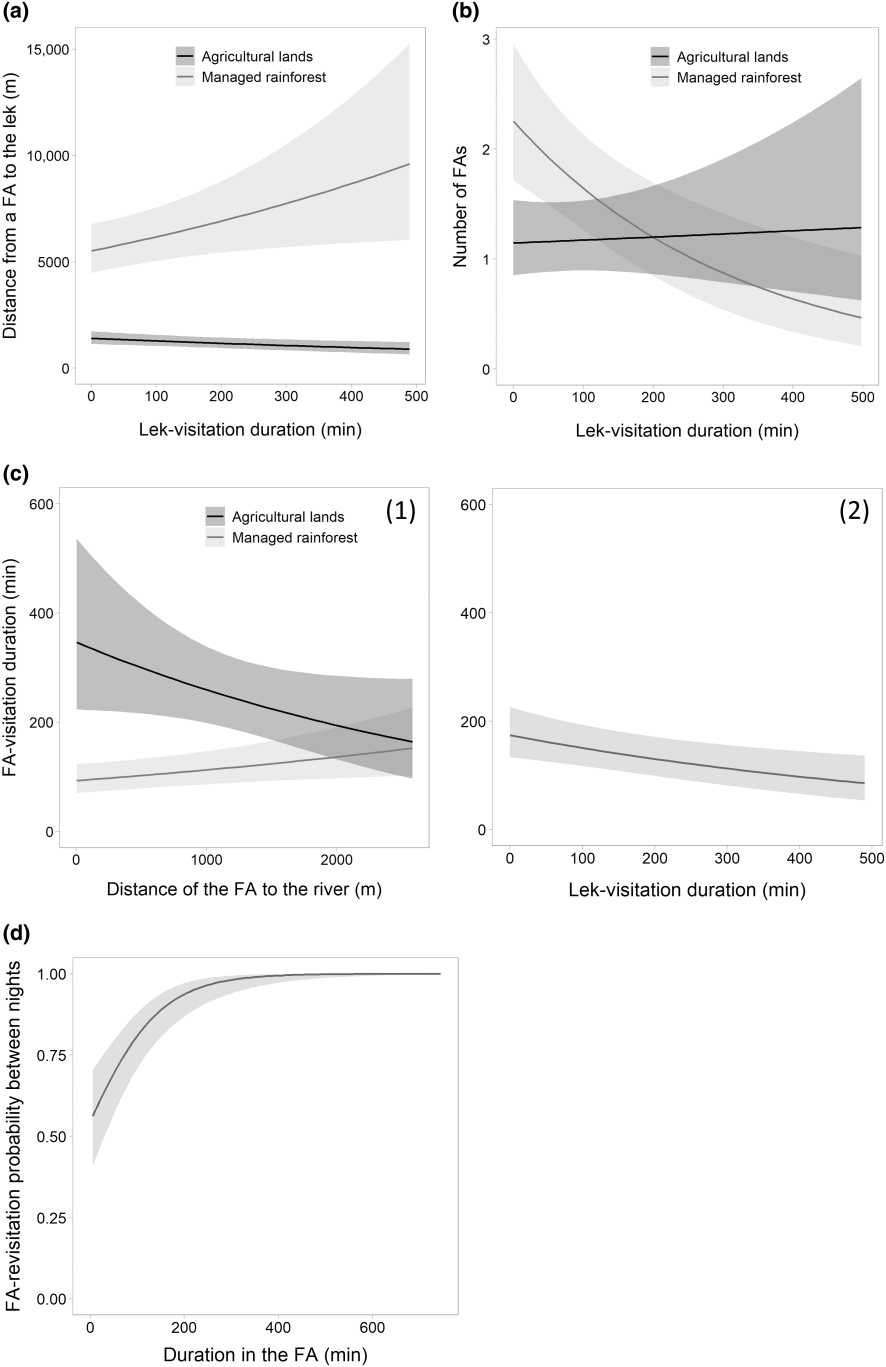
Estimation (with 95% CI; *n* = 16 individuals) of (a) the distance between a foraging area (FA) used by a bat a given night and the lek site (in m), according to the interaction between the habitat type of the FA and the total duration spent in the lek by the bat during that night, (b) the number of foraging areas (FAs) visited during the night by a bat, according to the interaction between the habitat type of the FA and the total duration spent in the lek by the bat during that night, (c) the total duration spent in a foraging area (FA) during the night by a bat (in min), according to (1) the interaction between the habitat type of the FA and the distance of the FA to the river (in m), and (2) the total duration spent in the lek during the night by the bat and (d) the probability of a bat revisiting a foraging area (FA) in the following 48 h, according to the total duration spent by the bat in that FA during the night (in min).

The model retained to explain the variation in the distance of the FAs used by a bat from the lek included the interaction effect between the habitat type of the FAs and the time spent in the lek during that night (ΔAICc of the following model = 13.54; Figure 4a). More concretely, bats visited FAs in the managed rainforest further from the lek when they spent a longer time therein, whereas they visited FAs in agricultural lands slightly closer.

Furthermore, the number of FAs used by a bat in the managed rainforest during the night was influenced by the same predictors (interaction effect; ΔAICc of the following model = 24.61; Figure 4b). Bats visited fewer FAs in the managed rainforest when they spend a longer time in the lek, whereas they visited a relative constant number in agricultural land. Bats visited a higher number of FAs in forest in comparison to agricultural lands for relatively short lek‐visitation durations.

The FA‐visitation duration by a bat during the night was influenced by the lek‐visitation duration during that night and by the interaction between the habitat type and the distance to the river of the FA (ΔAICc of the following model = 4.99; Figure 4c). Bats spent less time in FAs when they spend more time in the lek during the night. Their time spent in FAs of agricultural land also decreased with the distance to the river, whereas it slightly increased for FAs located in the managed rainforest. Overall, bats spent more time in FAs in agricultural lands.

Finally, the probability of a bat revisiting a given FA for two consecutive nights increased with the duration previously spent in that FA (ΔAICc of the following model = 1.94, ωAICc = 0.48; following ωAICc = 0.18; Figure 4d).

## 4 DISCUSSION

### 4.1 Human‐induced foraging pattern of bats

The combination of results related to foraging‐habitat selection and foraging‐behavior patterns of *H. monstrosus* males allowed us to better understand the foraging tendencies of the population studied. Individuals were more likely to forage in agricultural lands than in rainforest during the study period (Figure 2). Since our method accounted for the fact that bats were likely to select areas close to the lek, this pattern was not linked to the proximity of agricultural lands to the lek (Figure 1), but rather to foraging‐resources types. This result advocates for the presence of particularly abundant and attractive food resources in agricultural lands, such as *Musanga cecropioides*, plus additional suitable fruiting species (e.g., *Cissus dinklagei*, *Ficus* spp., *Macaranga* spp.; Appendix S2). Furthermore, bats spent more time in their foraging area in agricultural lands compared to forest during a night (Figure 4c). Overall, since bats did not visit a higher number of agricultural than forest foraging areas per night (it depends on their lek‐visitation duration; Figure 4b), the preference for agricultural lands is associated with a longer time spent in these agricultural areas. The marginal value theorem suggests that staying longer in higher quality patches may be a way for animals to optimize their food intake rate (Charnov, 1976). According to this optimal foraging theory, bats may benefit from remaining longer in agricultural areas due to their overall quality (i.e., abundance, diversity, distribution, and renewable rate of food resources).

In addition, bats preferred to forage in the vicinity of the rivers (Figure 2), an expected pattern in fruit bats species under natural condition (e.g., preserved forest; Mildenstein et al., 2005) due to the presence of specific hydrophilic fruiting tree species that provide food for bats. A field prospection confirmed that a key fruiting tree species (*Ficus mucuso)* was present in both habitats of the study area, and was significantly distributed closer to watercourses than randomly (Appendix S1). In the rainforest, areas located beyond 2 km from watercourses had a very low probability to be selected (Figure 2; maximum distance to the river being 5.36 km). This result supports the hypothesis of attractive food resources aggregated along rivers, despite a slightly visit‐duration increase for foraging areas distant to the river in comparison to the closest ones (Figure 4c1). In agricultural lands, the probability of selection (Figure 2) and the total duration spent in a foraging area by a bat during the night (Figure 4c1) strongly decreased with the distance to the river. Further investigations are needed to specify how the distribution of specific hydrophilic tree species providing food for bats may explain these patterns.

Overall, specific locations visited for longer periods by a given infected bat (i.e., mainly agricultural lands located close to the river) would increase the probability of contamination of those locations by potential pathogens hosted by this bat. Most viruses, including Ebola, probably do not survive outside their host over a few days in tropical forests (Nikiforuk et al., 2017). Given that the probability of a bat revisiting a foraging area in the following 48 h increased with the previous time spent in that area (Figure 4d), higher accumulation and longer periods of pathogen presence are also expected. However, short‐term longitudinal data on the viral excretion pattern at the individual level suggests intermittent viral excretion (Middleton et al., 2007; Suu‐Ire et al., 2018), inter‐individual variation (Schuh et al., 2017; Suu‐Ire et al., 2018), and is still limited to a few virus species inoculated to bats in laboratories. Further epidemiological studies would be required to estimate whether revisitation pattern may influence pathogen accumulation and persistence in these locations. The lack of effect of the other predictors on foraging area revisitation (i.e., habitat type and the distance to the river) is unexpected and deserves further investigations. For instance, overall food quality differences between habitats may be offset by other habitat quality features (e.g., human disturbance or global predation risk; Gül & Griffen, 2020).

### 4.2 Relationship between lek‐mating, roosting, and foraging behaviors

Studied bats have a higher probability to visit the lek site, and spent more time therein during the night with decreasing distance between their roost and the lek (Figure 3). Such a pattern likely results from a strategy limiting energy and time costs devoted to flights for the benefit of the mating activities of males, as the presence and time spent at the lek being known to increase individual breeding success (Vervoort & Kempenaers, 2019). Since no visit to another lek was identified during the study period, we suggest that a strong connectivity exists between males from a subpopulation centered within about a 10 km radius from the lek site (very low visitation probability beyond this distance; Figure 3a). Such use of a central place may promote local transmission and spread of infectious agents, as suggested in colonial breeding seabirds (McCoy et al., 2016) or insectivorous bat species (Webber et al., 2016). However, lek‐switch behaviors were possibly not recorded when individuals moved and remained out of the area covered by the reception antennas (seven among the 28 individuals studied), and we cannot exclude that some males play a role in inter‐connecting subpopulations (probably also females and juveniles; see Bradbury, 1977). Males may additionally change their roosting‐site location during the breeding period (based on the exploration of GPS data), likely adjusting that site selection according to their very recent or future mating investment in the lek.

In many animal species, foraging and breeding movements are interrelated to optimize survival and reproductive success (Geary et al., 2020; Staniland et al., 2007). An expected consequence in lek‐mating species is a reduction of overall time spent foraging for the benefit of mating activities (Cowles & Gibson, 2014). Our bats followed this pattern since foraging time decreased at the foraging‐area scale for active mating males (Figure 4c2). We found that most of these bats visited fewer foraging areas in forest, while the number of foraging areas visited in agricultural lands was constant (Figure 4b). This strategy may follow an exploitation‐exploration trade‐off based on the internal metabolic state (Corrales‐Carvajal et al., 2016) which led individuals to reduce travel time between areas and optimize food intake by selecting the most profitable ones (i.e., agricultural lands). Another strategy to reduce energetic costs related to travel is to forage closer to the central place (Pyke, 1984), as shown by our bats for agricultural areas (Figure 4a). Since an opposite result was found for forest areas (Figure 4a), and consistently with other central place foragers (Bruun & Smith, 2003; Staniland et al., 2007), we hypothesize that bats may travel a longer distance to reach specific profitable foraging areas. An additional analysis supports this hypothesis: bats that spent more time in the lek visited foraging areas closer to the river, which could be located far from the lek (i.e., expected high quality; Appendix S3 and Figure 1). Overall, these results suggest high inter‐individual variations in lek‐mating associated movements and provide high‐resolution data to model contact network in an epidemiological framework (Craft & Caillaud, 2011).

We hypothesized that the foraging attractiveness of both agricultural lands and watercourses previously discussed for males likely influenced the establishment of the lek area nearby. Lek establishment by males is likely also linked to the probability of encountering females locally (Westcott, 1994). Unfortunately, we did not collect GPS data on females (i.e., individual weight issues), leading to a limitation to characterize mating and foraging behaviors of this bat population. More specifically, given the variation in the mating activities and constraints between sexes (e.g., females visiting several leks established by males to mate, and specific physiological investment during breeding; Lebigre et al., 2013; Storch, 1997), at least slight differences in lek‐visitation patterns may be expected. In addition, sex‐specific foraging strategies and resources partitioning may occur in this species, as often observed in bats (Maynard et al., 2019). However, we observed females feeding with males on trees such as *M. cercopoides* and *Ficus* spp. in the vicinity of villages, suggesting some similarities in foraging habitat use and selection patterns between sexes. The consideration of females nevertheless remains to be explored.

### 4.3 Global implication in an epidemiological framework

Given local human activities in the region (e.g., agricultural work, hunting, and gold mining into riverbeds), space use patterns of bats during the mating period clearly indicated multiple potential direct (e.g., hunting) or indirect (e.g., contaminated food resources) human‐bat contacts. Furthermore, the proximity of leks with human settlements could disturb the reproduction of the species and lead to conservation impacts. Also, places of interest for bats near watercourses may overlap the space used for human activities linked to water (e.g., fishing, washing, and collection of drinking water). Because water‐related environments are typically considered as important transmission routes of infectious diseases (e.g., via deposition of urines, feces, or saliva; Hurst, 2018), future studies should particularly consider the surveillance of viral pathogens in watercourses both within and near lek sites. Since Ebola virus dynamics involve multi‐species hosts (e.g., bats, primates, other wild and domestic mammals; Weingartl et al., 2013), investigating contact networks between species near fruiting trees within attractive foraging areas highlighted by our work could complementarily enhance the knowledge about Ebola virus ecology (Caron et al., 2018). Indeed, field visits on foraging patches used by bats in agricultural lands revealed a strong preference for native tree species, which are also a suitable food for other wild and domestic animals (Appendix S2).

## 5 CONCLUSIONS

Overall, *H. Monstrosus* is a generalist frugivorous species that may benefit from at least a low level of human‐modified habitats. Replication of such studies in both sexes, in contrasted environments (i.e., along a gradient of human‐modified landscape) or in seasons where native fruiting resources are scarce (i.e., June to August in similar regions; Adamescu et al., 2018; Gautier‐Hion et al., 1985), may contribute to understand how a species typically living in tropical forest progressively adapt or not to human‐modified environments and interact with humans. Similarly, the ecology of the viruses hosted by these species may be impacted by these modifications and promote or not viral emergence in humans and their associated species.

## AUTHOR CONTRIBUTIONS

**Elodie Schloesing:** Conceptualization (equal); data curation (lead); formal analysis (lead); methodology (lead); writing – original draft (lead); writing – review and editing (equal). **Alexandre Caron:** Conceptualization (equal); funding acquisition (supporting); methodology (equal); project administration (equal); supervision (lead); writing – review and editing (equal). **Rémi Chambon:** Conceptualization (equal); data curation (lead); formal analysis (lead); methodology (lead); writing – original draft (lead); writing – review and editing (lead). **Nicolas Courbin:** Formal analysis (equal); methodology (supporting); writing – review and editing (equal). **Morgane Labadie:** Data curation (equal); methodology (supporting); project administration (equal); writing – review and editing (equal). **Roch Nina:** Data curation (supporting); project administration (supporting); writing – review and editing (supporting). **Frida Mouiti Mbadinga:** Data curation (supporting); project administration (supporting); writing – review and editing (supporting). **Wilfrid Ngoubili:** Data curation (supporting); writing – review and editing (supporting). **Danfici Sandiala:** Data curation (supporting); writing – review and editing (supporting). **N'Kaya Tobi:** Conceptualization (supporting); project administration (equal); writing – review and editing (supporting). **Mathieu Bourgarel:** Conceptualization (equal); funding acquisition (lead); methodology (supporting); project administration (lead); supervision (equal); writing – review and editing (equal). **Hélène M De Nys:** Conceptualization (equal); funding acquisition (equal); methodology (equal); project administration (lead); supervision (equal); writing – review and editing (equal). **Julien Cappelle:** Conceptualization (equal); data curation (equal); funding acquisition (equal); methodology (equal); project administration (equal); supervision (equal); writing – review and editing (equal).

## FUNDING INFORMATION

This work was supported by the European Commission (FOOD/2016/379–660, EBOSURSY project, Ph.D. grant to ES).

## CONFLICT OF INTEREST STATEMENT

The authors declare that they have no conflict of interest.

## Supporting information


Appendix S1–S5

## Data Availability

The data analyzed during the current study will be made available after a 1‐year embargo in the Movebank Data Repository, https://doi.org/10.5441/001/1.278 (Schloesing et al., 2024).

## References

[ece310240-bib-0001] Adamescu, G. S. , Plumptre, A. J. , Abernethy, K. A. , Polansky, L. , Bush, E. R. , Chapman, C. A. , Shoo, L. P. , Fayolle, A. , Janmaat, K. R. L. , Robbins, M. M. , Ndangalasi, H. J. , Cordeiro, N. J. , Gilby, I. C. , Wittig, R. M. , Breuer, T. , Hockemba, M. B. N. , Sanz, C. M. , Morgan, D. B. , Pusey, A. E. , … Beale, C. M. (2018). Annual cycles are the most common reproductive strategy in African tropical tree communities. Biotropica, 50(3), 418–430. 10.1111/btp.12561

[ece310240-bib-0002] Alcock, J. (2013). Animal behavior: An evolutionary approach (10th ed.). Sinauer Associates.

[ece310240-bib-0003] Baudel, H. , Nys, H. D. , Ngole, E. M. , Peeters, M. , & Desclaux, A. (2019). Understanding Ebola virus and other zoonotic transmission risks through human–bat contacts: Exploratory study on knowledge, attitudes and practices in southern Cameroon. Zoonoses and Public Health, 66(3), 288–295. 10.1111/zph.12563 30677236 PMC7165775

[ece310240-bib-0004] Bellón, B. , Bégué, A. , Lo Seen, D. , de Almeida, C. , & Simões, M. (2017). A remote sensing approach for regional‐scale mapping of agricultural land‐use systems based on NDVI time series. Remote Sensing, 9(6), 600. 10.3390/rs9060600

[ece310240-bib-0005] Benavides, J. , Walsh, P. D. , Meyers, L. A. , Raymond, M. , & Caillaud, D. (2012). Transmission of infectious diseases En route to habitat hotspots. PLoS ONE, 7(2), e31290. 10.1371/journal.pone.0031290 22363606 PMC3282722

[ece310240-bib-0006] Benhamou, S. (2011). Dynamic approach to space and habitat use based on biased random bridges. PLoS One, 6(1), e14592. 10.1371/journal.pone.0014592 21297869 PMC3027622

[ece310240-bib-0007] Boulinier, T. , Kada, S. , Ponchon, A. , Dupraz, M. , Dietrich, M. , Gamble, A. , Bourret, V. , Duriez, O. , Bazire, R. , Tornos, J. , Tveraa, T. , Chambert, T. , Garnier, R. , & McCoy, K. D. (2016). Migration, prospecting, dispersal? What host movement matters for infectious agent circulation? Integrative and Comparative Biology, 56(2), 330–342. 10.1093/icb/icw015 27252195

[ece310240-bib-0008] Boyce, M. S. , Vernier, P. R. , Nielsen, S. E. , & Schmiegelow, F. K. A. (2002). Evaluating resource selection functions. Ecological Modelling, 157(2–3), 281–300. 10.1016/S0304-3800(02)00200-4

[ece310240-bib-0009] Bradbury, J. W. (1977). Lek mating behavior in the hammer‐headed bat. Zeitschrift für Tierpsychologie, 45(3), 225–255. 10.1111/j.1439-0310.1977.tb02120.x

[ece310240-bib-0010] Brown, D. D. , LaPoint, S. , Kays, R. , Heidrich, W. , Kümmeth, F. , & Wikelski, M. (2012). Accelerometer‐informed GPS telemetry: Reducing the trade‐off between resolution and longevity. Wildlife Society Bulletin, 36(1), 139–146. 10.1002/wsb.111

[ece310240-bib-0011] Bruun, M. , & Smith, H. G. (2003). Landscape composition affects habitat use and foraging flight distances in breeding European starlings. Biological Conservation, 114(2), 179–187. 10.1016/S0006-3207(03)00021-1

[ece310240-bib-0012] Calisher, C. H. , Childs, J. E. , Field, H. E. , Holmes, K. V. , & Schountz, T. (2006). Bats: Important reservoir hosts of emerging viruses. Clinical Microbiology Reviews, 19(3), 531–545. 10.1128/CMR.00017-06 16847084 PMC1539106

[ece310240-bib-0013] Cappelle, J. , Hoem, T. , Hul, V. , Furey, N. , Nguon, K. , Prigent, S. , Dupon, L. , Ken, S. , Neung, C. , Hok, V. , Pring, L. , Lim, T. , Bumrungsri, S. , Duboz, R. , Buchy, P. , Ly, S. , Duong, V. , Tarantola, A. , Binot, A. , & Dussart, P. (2020). Nipah virus circulation at human–bat interfaces, Cambodia. Bull World Health Organization, 98(8), 539–547. 10.2471/BLT.20.254227 PMC7411325 32773899

[ece310240-bib-0014] Caron, A. , Bourgarel, M. , Cappelle, J. , Liégeois, F. , De Nys, H. M. , & Roger, F. (2018). Ebola virus maintenance: If not (only) bats, what Else? Viruses, 10, 549. 10.3390/v10100549 30304789 PMC6213544

[ece310240-bib-0015] Carpenter, R. E. (1986). Flight physiology of intermediate‐sized fruit bats (Pteropodidae). The Journal of Experimental Biology, 120(1), 79–103. 10.1242/jeb.120.1.79

[ece310240-bib-0016] Charnov, E. L. (1976). Optimal foraging, the marginal value theorem. Theoretical Population Biology, 9(2), 129–136. 10.1016/0040-5809(76)90040-X 1273796

[ece310240-bib-0017] Corrales‐Carvajal, V. M. , Faisal, A. A. , & Ribeiro, C. (2016). Internal states drive nutrient homeostasis by modulating exploration‐exploitation trade‐off. eLife, 5, e19920. 10.7554/eLife.19920 27770569 PMC5108593

[ece310240-bib-0018] Cowles, S. A. , & Gibson, R. M. (2014). Displaying to females may lower male foraging time and vigilance in a lekking bird. The Auk, 132(1), 82–91. 10.1642/AUK-14-67.1

[ece310240-bib-0019] Craft, M. E. , & Caillaud, D. (2011). Network models: An underutilized tool in wildlife epidemiology? Interdisciplinary Perspectives on Infectious Diseases, 2011, e676949. 10.1155/2011/676949 PMC3063006 21527981

[ece310240-bib-0020] Crichton, E. G. , & Krutzsch, P. H. (Eds.). (2000). Reproductive biology of bats. Academic Press.

[ece310240-bib-0021] de Nys, H. M. , Kingebeni, P. M. , Keita, A. K. , Butel, C. , Thaurignac, G. , Villabona‐Arenas, C.‐J. , Lemarcis, T. , Geraerts, M. , Vidal, N. , Esteban, A. , Bourgarel, M. , Roger, F. , Leendertz, F. , Diallo, R. , Ndimbo‐Kumugo, S. P. , Nsio‐Mbeta, J. , Tagg, N. , Koivogui, L. , Toure, A. , … Peeters, M. (2018). Survey of Ebola viruses in frugivorous and insectivorous bats in Guinea, Cameroon, and The Democratic Republic of the Congo, 2015–2017. Emerging Infectious Diseases, 24(12), 2228–2240. 10.3201/eid2412.180740 30307845 PMC6256401

[ece310240-bib-0022] Dougherty, E. R. , Seidel, D. P. , Carlson, C. J. , Spiegel, O. , & Getz, W. M. (2018). Going through the motions: Incorporating movement analyses into disease research. Ecology Letters, 21(4), 588–604. 10.1111/ele.12917 29446237

[ece310240-bib-0023] Dürr, S. , & Ward, M. P. (2014). Roaming behaviour and home range estimation of domestic dogs in aboriginal and Torres Strait islander communities in northern Australia using four different methods. Preventive Veterinary Medicine, 117(2), 340–357. 10.1016/j.prevetmed.2014.07.008 25096735

[ece310240-bib-0024] Epstein, J. H. , Zambriski, J. A. , Rostal, M. K. , Heard, D. J. , & Daszak, P. (2011). Comparison of intravenous Medetomidine and Medetomidine/ketamine for immobilization of free‐ranging variable flying foxes (*Pteropus hypomelanus*). PLoS ONE, 6(10), e25361. 10.1371/journal.pone.0025361 22065987 PMC3204968

[ece310240-bib-0025] Fernandez‐Gimenez, M. , & Allen‐Diaz, B. (2001). Vegetation change along gradients from water sources in three\011\011grazed Mongolian ecosystems. Plant Ecology Former Vegetative, 157(1), 101–118. 10.1023/A:1014519206041

[ece310240-bib-0026] Furmankiewicz, J. , & Kucharska, M. (2009). Migration of bats along a large River Valley in southwestern Poland. Journal of Mammalogy, 90(6), 1310–1317. 10.1644/09-MAMM-S-099R1.1

[ece310240-bib-0027] Gautier‐Hion, A. , Duplantier, J.‐M. , Emmons, L. , Feer, F. , Heckestweiler, P. , Moungazi, A. , Quris, R. , & Sourd, C. (1985). Coadaptation entre rythmes de fructification et frugivorie en forêt tropicale humide du Gabon: Mythe ou réalité. Revue d'Écologie (La Terre et La Vie), 40(4), 405–434.

[ece310240-bib-0028] Gautier‐Hion, A. , & Michaloud, G. (1989). Are figs always keystone resources for tropical frugivorous vertebrates? A test in gabon. Ecology, 70(6), 1826–1833. 10.2307/1938115

[ece310240-bib-0029] Geary, B. , Leberg, P. L. , Purcell, K. M. , Walter, S. T. , & Karubian, J. (2020). Breeding Brown pelicans improve foraging performance as energetic needs rise. Scientific Reports, 10, 1686. 10.1038/s41598-020-58528-z 32015412 PMC6997155

[ece310240-bib-0030] Grundy, E. , Jones, M. W. , Laramee, R. S. , Wilson, R. P. , & Shepard, E. L. C. (2009). Visualisation of sensor data from animal movement. Computer Graphics Forum, 28(3), 815–822. 10.1111/j.1467-8659.2009.01469.x

[ece310240-bib-0031] Gül, M. R. , & Griffen, B. D. (2020). Diet, energy storage, and reproductive condition in a bioindicator species across beaches with different levels of human disturbance. Ecological Indicators, 117, 106636. 10.1016/j.ecolind.2020.106636

[ece310240-bib-0032] Hurst, C. J. (2018). Understanding and estimating the risk of waterborne infectious disease associated with drinking water. In C. J. Hurst (Ed.), The connections between ecology and infectious disease (pp. 59–114). Springer International Publishing. 10.1007/978-3-319-92373-4_3

[ece310240-bib-0033] Johnson, D. S. , Hooten, M. B. , & Kuhn, C. E. (2013). Estimating animal resource selection from telemetry data using point process models. The Journal of Animal Ecology, 82(6), 1155–1164. 10.1111/1365-2656.12087 23800202

[ece310240-bib-0034] Kareiva, P. , & Odell, G. (1987). Swarms of predators exhibit ‘Preytaxis’ if individual predators use area‐restricted search. The American Naturalist, 130(2), 233–270.

[ece310240-bib-0035] Lebigre, C. , Alatalo, R. V. , & Siitari, H. (2013). Physiological costs enforce the honesty of lek display in the black grouse (*Tetrao tetrix*). Oecologia, 172(4), 983–993. 10.1007/s00442-012-2548-9 23266713

[ece310240-bib-0036] Leroy, E. M. , Epelboin, A. , Mondonge, V. , Pourrut, X. , Gonzalez, J.‐P. , Muyembe‐Tamfum, J.‐J. , & Formenty, P. (2009). Human Ebola outbreak resulting from direct exposure to fruit bats in Luebo, Democratic Republic of Congo, 2007. Vector‐Borne Zoonotic Disease, 9(6), 723–728. 10.1089/vbz.2008.0167 19323614

[ece310240-bib-0037] Martin, J. , Tolon, V. , Moorter, B. , Basille, M. , & Calenge, C. (2009). On the use of telemetry in habitat selection studies. In D. Barculo & J. Daniels (Eds.), Telemetry: Research, technology and applications (pp. 37–55). Nova Publishers.

[ece310240-bib-0038] Maynard, L. D. , Ananda, A. , Sides, M. F. , Burk, H. , & Whitehead, S. R. (2019). Dietary resource overlap among three species of frugivorous bat in Costa Rica. Journal of Tropical Ecology, 35(4), 165–172. 10.1017/S0266467419000129

[ece310240-bib-0039] McClintock, B. T. , & Michelot, T. (2018). momentuHMM: r package for generalized hidden Markov models of animal movement. Methods in Ecology and Evolution, 9(6), 1518–1530. 10.1111/2041-210X.12995

[ece310240-bib-0040] McCoy, K. D. , Dietrich, M. , Jaeger, A. , Wilkinson, D. A. , Bastien, M. , Lagadec, E. , Boulinier, T. , Pascalis, H. , Tortosa, P. , le Corre, M. , Dellagi, K. , & Lebarbenchon, C. (2016). The role of seabirds of the Iles Eparses as reservoirs and disseminators of parasites and pathogens. Acta Oecologica, 72, 98–109. 10.1016/j.actao.2015.12.013 32288503 PMC7128210

[ece310240-bib-0041] McEvoy, J. F. , Kishbaugh, J. C. , Valitutto, M. T. , Aung, O. , Tun, K. Y. N. , Win, Y. T. , Maw, M. T. , Thein, W. Z. , Win, H. H. , Chit, A. M. , Vodzak, M. E. , & Murray, S. (2021). Movements of Indian flying fox in Myanmar as a guide to human‐bat Interface sites. EcoHealth, 18(2), 204–216. 10.1007/s10393-021-01544-w 34448977 PMC8390844

[ece310240-bib-0042] Middleton, D. J. , Morrissy, C. J. , van der Heide, B. M. , Russell, G. M. , Braun, M. A. , Westbury, H. A. , Halpin, K. , & Daniels, P. W. (2007). Experimental Nipah virus infection in Pteropid bats (*Pteropus poliocephalus*). Journal of Comparative Pathology, 136(4), 266–272. 10.1016/j.jcpa.2007.03.002 17498518

[ece310240-bib-0043] Mildenstein, T. , Tanshi, I. , & Racey, P. A. (2016). Exploitation of bats for Bushmeat and medicine. In C. C. Voigt & T. Kingston (Eds.), Bats in the Anthropocene: Conservation of bats in a changing world (pp. 325–375). Springer International Publishing. 10.1007/978-3-319-25220-9_12

[ece310240-bib-0044] Mildenstein, T. L. , Stier, S. C. , Nuevo‐Diego, C. E. , & Mills, L. S. (2005). Habitat selection of endangered and endemic large flying‐foxes in Subic Bay, Philippines. Biological Conservation, 126(1), 93–102. 10.1016/j.biocon.2005.05.001

[ece310240-bib-0045] Mollentze, N. , & Streicker, D. G. (2020). Viral zoonotic risk is homogenous among taxonomic orders of mammalian and avian reservoir hosts. Proceedings of the National Academy of Sciences, 117(17), 9423–9430. 10.1073/pnas.1919176117 PMC7196766 32284401

[ece310240-bib-0046] Monsarrat, S. , Benhamou, S. , Sarrazin, F. , Bessa‐Gomes, C. , Bouten, W. , & Duriez, O. (2013). How predictability of feeding patches affects home range and foraging habitat selection in avian social scavengers? PLoS One, 8(1), e53077. 10.1371/journal.pone.0053077 23301024 PMC3536817

[ece310240-bib-0047] Muff, S. , Signer, J. , & Fieberg, J. (2020). Accounting for individual‐specific variation in habitat‐selection studies: Efficient estimation of mixed‐effects models using Bayesian or frequentist computation. The Journal of Animal Ecology, 89(1), 80–92. 10.1111/1365-2656.13087 31454066

[ece310240-bib-0048] Nading, A. M. (2013). Humans, animals, and health: From ecology to entanglement. Environmental Sociology, 4(1), 60–78. 10.3167/ares.2013.040105

[ece310240-bib-0049] Niamien, C. , Yaokokoré‐Béibro, H. , Koné, I. , & N'goran, K. (2010). Données préliminaires Sur l'écologie des chauves‐Souris frugivores de la commune du plateau (Abidjan, Côte D'ivoire). The Science of Nature, 7(1), 21–30. 10.4314/scinat.v7i1.59915

[ece310240-bib-0050] Nikiforuk, A. M. , Cutts, T. A. , Theriault, S. S. , & Cook, B. W. M. (2017). Challenge of liquid stressed protective materials and environmental persistence of Ebola virus. Scientific Reports, 7(1), 4388. 10.1038/s41598-017-04137-2 28663587 PMC5491502

[ece310240-bib-0051] Olson, S. H. , Bounga, G. , Ondzie, A. , Bushmaker, T. , Seifert, S. N. , Kuisma, E. , Taylor, D. W. , Munster, V. J. , & Walzer, C. (2019). Lek‐associated movement of a putative ebolavirus reservoir, the hammer‐headed fruit bat (*Hypsignathus monstrosus*), in northern republic of Congo. PLoS One, 14(10), e0223139. 10.1371/journal.pone.0223139 31574111 PMC6772046

[ece310240-bib-0052] O'Mara, M. T. , Wikelski, M. , & Dechmann, D. K. N. (2014). 50 years of bat tracking: Device attachment and future directions. Methods in Ecology and Evolution, 5(4), 311–319. 10.1111/2041-210X.12172

[ece310240-bib-0053] Patin, R. , Etienne, M.‐P. , Lebarbier, E. , Chamaillé‐Jammes, S. , & Benhamou, S. (2020). Identifying stationary phases in multivariate time series for highlighting behavioural modes and home range settlements. The Journal of Animal Ecology, 89(1), 44–56. 10.1111/1365-2656.13105 31539165

[ece310240-bib-0054] Pyke, G. (1984). Optimal foraging theory: A critical review. Annual Review of Ecology and Systematics, 15(1), 523–575. 10.1146/annurev.ecolsys.15.1.523

[ece310240-bib-0055] Reaney, L. T. (2007). Foraging and mating opportunities influence refuge use in the fiddler crab, Uca Mjoebergi. Animal Behaviour, 73(4), 711–716. 10.1016/j.anbehav.2006.05.022

[ece310240-bib-0056] Rugarabamu, S. , Mboera, L. , Rweyemamu, M. , Mwanyika, G. , Lutwama, J. , Paweska, J. , & Misinzo, G. (2020). Forty‐two years of responding to Ebola virus outbreaks in sub‐Saharan Africa: A review. BMJ Global Health, 5(3), e001955. 10.1136/bmjgh-2019-001955 PMC7061886 32201623

[ece310240-bib-0057] Rydell, J. , Bach, L. , Bach, P. , Diaz, L. G. , Furmankiewicz, J. , Hagner‐Wahlsten, N. , Kyheröinen, E.‐M. , Lilley, T. , Masing, M. , Meyer, M. M. , Ptersons, G. , Šuba, J. , Vasko, V. , Vintulis, V. , & Hedenström, A. (2014). Phenology of migratory bat activity across the Baltic Sea and the south‐eastern North Sea. Acta Chiropterologica, 16(1), 139–147. 10.3161/150811014X683354

[ece310240-bib-0058] Schloesing, E. , Caron, A. , Chambon, R. , Courbin, N. , Labadie, M. , Nina, R. , Mouiti Mbadinga, F. , Ngoubili, W. , Sandiala, D. , & ‐Tobi, N.'. K. (2024). Data from: Foraging and mating behaviors of Hypsignathus monstrosus at the bat‐human interface in a central African rainforest. Movebank Data Repository. 10.5441/001/1.278 PMC10329260 37424939

[ece310240-bib-0059] Schloesing, E. , Chambon, R. , Tran, A. , Choden, K. , Ravon, S. , Epstein, J. H. , Hoem, T. , Furey, N. , Labadie, M. , Bourgarel, M. , de Nys, H. M. , Caron, A. , & Cappelle, J. (2020). Patterns of foraging activity and fidelity in a southeast Asian flying fox. Movement Ecology, 8(1), 46. 10.1186/s40462-020-00232-8 33292573 PMC7652672

[ece310240-bib-0060] Schoener, T. W. (1971). Theory of feeding strategies. Annual Review of Ecology and Systematics, 2(1), 369–404. 10.1146/annurev.es.02.110171.002101

[ece310240-bib-0061] Schuh, A. J. , Amman, B. R. , Jones, M. E. B. , Sealy, T. K. , Uebelhoer, L. S. , Spengler, J. R. , Martin, B. E. , Coleman‐McCray, J. A. D. , Nichol, S. T. , & Towner, J. S. (2017). Modelling filovirus maintenance in nature by experimental transmission of Marburg virus between Egyptian rousette bats. Nature Communications, 8(1), 14446. 10.1038/ncomms14446 PMC5316840 28194016

[ece310240-bib-0062] Shaffer, S. A. , Costa, D. P. , & Weimerskirch, H. (2003). Foraging effort in relation to the constraints of reproduction in free‐ranging albatrosses: *Foraging effort of free‐ranging albatrosses* . Functional Ecology, 17(1), 66–74. 10.1046/j.1365-2435.2003.00705.x

[ece310240-bib-0063] Sharma, V. , Sulochana, K. , Kumar, R. , Yadav, J. P. , & Samander, K. (2019). Emerging trends of Nipah virus: A review. Reviews in Medical Virology, 29(1), e2010. 10.1002/rmv.2010 30251294 PMC7169151

[ece310240-bib-0064] Sikes, R. S. , & Gannon, W. L. (2011). Guidelines of the American Society of Mammalogists for the use of wild mammals in research. Journal of Mammalogy, 92(1), 235–253. 10.1644/10-MAMM-F-355.1 PMC5909806 29692469

[ece310240-bib-0065] Sikes, R. S. , & the Animal Care and Use Committee of the American Society of Mammalogists . (2016). 2016 Guidelines of the American Society of Mammalogists for the use of wild mammals in research and education. Journal of Mammalogy, 97(3), 663–688. 10.1093/jmammal/gyw078 29692469 PMC5909806

[ece310240-bib-0066] Staniland, I. J. , Boyd, I. L. , & Reid, K. (2007). An energy–distance trade‐off in a central‐place forager, the Antarctic fur seal (*Arctocephalus gazella*). Marine Biology, 152(2), 233–241. 10.1007/s00227-007-0698-9

[ece310240-bib-0067] Storch, I. (1997). Male territoriality, female range use, and spatial organisation of capercaillie *Tetrao urogallus* leks. Wildlife Biology, 3(1), 149–161. 10.2981/wlb.1997.019

[ece310240-bib-0068] Suu‐Ire, R. , Begeman, L. , Banyard, A. C. , Breed, A. C. , Drosten, C. , Eggerbauer, E. , Freuling, C. M. , Gibson, L. , Goharriz, H. , Horton, D. L. , Jennings, D. , Kuzmin, I. V. , Marston, D. , Ntiamoa‐Baidu, Y. , Riesle Sbarbaro, S. , Selden, D. , Wise, E. L. , Kuiken, T. , Fooks, A. R. , … Cunningham, A. A. (2018). Pathogenesis of bat rabies in a natural reservoir: Comparative susceptibility of the straw‐colored fruit bat (*Eidolon helvum*) to three strains of Lagos bat virus. PLoS Neglected Tropical Diseases, 12(3), e0006311. 10.1371/journal.pntd.0006311 29505617 PMC5854431

[ece310240-bib-0069] Tanshi, I. (2016). IUCN red list of threatened species: Hypsignathus monstrosus. *IUCN Red List Threat Species* . https://www.iucnredlist.org/en

[ece310240-bib-0070] Uchii, K. , Telschow, A. , Minamoto, T. , Yamanaka, H. , Honjo, M. N. , Matsui, K. , & Kawabata, Z. (2011). Transmission dynamics of an emerging infectious disease in wildlife through host reproductive cycles. The ISME Journal, 5(2), 244–251. 10.1038/ismej.2010.123 20740025 PMC3105706

[ece310240-bib-0071] Vervoort, R. , & Kempenaers, B. (2019). Variation in lek attendance and copulation success of independent and satellite male ruffs Calidris pugnax. Ardea, 107(3), 303–320. 10.5253/arde.v107i3.a9

[ece310240-bib-0072] Waghiiwimbom, M. D. , Eric‐Moise, B. F. , Jules, A. P. , Aimé, T. K. J. , & Tamesse, J. L. (2020). Diversity and community structure of bats (Chiroptera) in the Centre region of Cameroon. African Journal of Ecology, 58(2), 211–226. 10.1111/aje.12692

[ece310240-bib-0073] Webber, Q. M. R. , Brigham, R. M. , Park, A. D. , Gillam, E. H. , O'Shea, T. J. , & Willis, C. K. R. (2016). Social network characteristics and predicted pathogen transmission in summer colonies of female big brown bats (*Eptesicus fuscus*). Behavioral Ecology and Sociobiology, 70(5), 701–712. 10.1007/s00265-016-2093-3

[ece310240-bib-0074] Weingartl, H. , Nfon, C. , & Kobinger, G. (2013). Review of Ebola virus infections in domestic animals. Developmental Biology, 135, 211–218. 10.1159/000178495 23689899

[ece310240-bib-0075] Westcott, D. (1994). Leks of leks: A role for hotspots in lek evolution? Proceedings of the Royal Society of London – Series B: Biological Sciences, 258, 281–286. 10.1098/rspb.1994.0174

[ece310240-bib-0076] Williams‐Guillén, K. , Olimpi, E. , Maas, B. , Taylor, P. J. , & Arlettaz, R. (2016). Bats in the anthropogenic matrix: Challenges and opportunities for the conservation of Chiroptera and their ecosystem Services in Agricultural Landscapes. In C. C. Voigt & T. Kingston (Eds.), Bats in the Anthropocene: Conservation of bats in a changing world (pp. 151–186). Springer International Publishing.

