# CORRIGENDUM

**DOI:** 10.1002/ece3.10350

**Published:** 2023-07-19

In the recent article by Schloesing et al. (2023), the authors would like to acknowledge that author “N'Kaya Tobi” was deleted in error in the original publication. The correct list of authors is as follows:

Elodie Schloesing| Alexandre Caron | Rémi Chambon| Nicolas Courbin| Morgane Labadie| Roch Nina | Frida Mouiti Mbadinga | Wilfrid Ngoubili |Danficy Sandiala | N'Kaya Tobi | Mathieu Bourgarel | Hélène M. De Nys | Julien Cappelle.

The online version has been corrected online.
