# Supplementary material for: Foraging and mating behaviors of Hypsignathus monstrosus at the bat‐human interface in a central African rainforest
Source: Ecol Evol. 2023 Jul 8;13(7):e10240. doi: 10.1002/ece3.10240 (PMC10329260; doi:10.1002/ece3.10240)
Supplement: Supplementary file 1 — Appendix S1–S5 [file ECE3-13-e10240-s001.docx]

## Foraging and mating behaviors of *Hypsignathus monstrosus* at the bat-human interface in a central African rainforest

**Appendix**

**Appendix S1:** Distribution of fruiting *Ficus mucuso* along a 40 km-long path.

**Appendix S2:** Occurrence of fruiting tree species in foraging patches of the managed rainforest and agricultural lands, and occurrence of bat guano near individual fruiting trees.

**Appendix S3:** Additional analysis about the variation on the mean distance between a foraging area used by a bat during the night and the river.

**Appendix S4:** Additional details on space used by bats.

**Appendix S5:** Model selection, results of retained models, and RSF model validation.

**Appendix S1**

**Materiel and methods**

Some fruiting trees consumed by bats are especially abundant along rivers in central Africa (e.g., *Ficus spp*.; Gautier-Hion and Michaloud 1989). To improve our knowledge about the distribution of such specific resources along rivers in the study region, we monitored the distribution of fruiting *Ficus mucuso* along a 40 km-long path – a road crossing the region (both the managed rainforest and agricultural lands) with a variable distance to rivers. An inventory was carried out once along the entire length of that path between the 9^th^and 12^th^ February 2020 (some consecutive portions were surveyed on consecutive days). More specifically, four trained observers were looking for fruiting *F. mucuso* over a distance of 50 m on each side of the path, from a vehicle in motion (speed: around 30 km/h). Half of the observers focused on the right of the path, and the two others on the left. As a result, 26 fruiting trees were recorded and their GPS locations were projected on a map (**Figure S3.1**).

We hypothesized that fruiting *F. mucuso* were distributed closer to the river than random. To test this assumption, we generated 26 random points (representing random locations of hypothetic trees) into the same area covered by the field observations (i.e. 40 km-long and 100 m-wide path; **Figure S3.1**). The distance between the river and each location (true and random) was calculated. The effect of the type of location (true and random) was tested on that distance, using a GLM (Gamma error distribution, log link function; significance level: α = 0.05). Pseudo R-squared was computed.

**Results**

The mean distance between the river and each location was significantly lower for true *F. mucuso* than random locations (χ^2^ = 9.4, df = 1, *P* < 0.01; R² = 0.14; **Figure S3.1**). Parameter estimates (lower and upper 95% confidence interval) were 6.70 (6.47; 6.91) for the intercept and 0.48 (0.18; 0.80) for the type of location. The ‘true location’ being the implicit reference level in the intercept component.


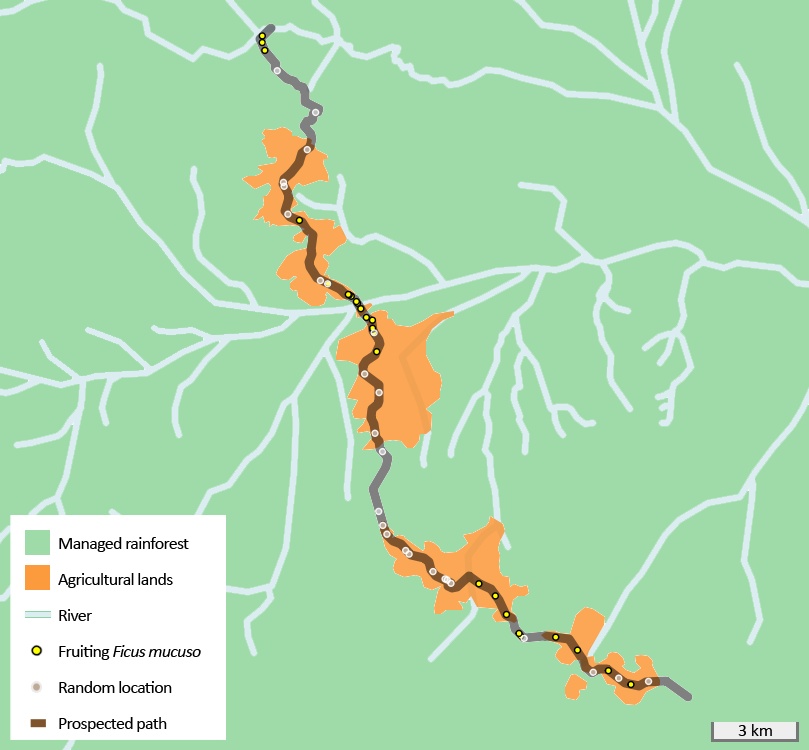


**Figure S3.1.** Distribution of fruiting *Ficus mucuso* along a 40 km path crossing the study region (surveyed once from 9^th^ to 12^th^ of February 2020), and 26 random locations used for the analysis presented above.

**Reference**

Gautier-Hion A, Michaloud G. 1989. Are Figs Always Keystone Resources for Tropical Frugivorous Vertebrates? A Test in Gabon. Ecology. 70(6):1826–1833. doi:10.2307/1938115.

**Appendix S2**

**Materials and methods**

A total of 61 foraging patches (selected from the GPS location of 23 bats) were visited once during the study period (17^th^ to 29^th^ of January 2020). All fruiting trees with ripe fruits were identified within a radius of 15 m from a focal location in the centre of the patch. Among these patches, 43 included fruiting trees and 18 were resting or feeding perches only (no fruiting trees). The presence of guano was visually searched near individual fruiting trees (indices of fruit consumption).

**Results**

The main fruiting species very likely consumed (assessed by the presence of fresh bat guano near at least one individual tree) at foraging patches we visited were: *Musanga cecropioides* (found in 41% of the patches in agricultural lands and 8% in the managed rainforest), *Ficus mucuso*, *Cissus dinklagei*, and *Macaranga spp.* (3-8% of patches, depending on tree species and habitat type; see **Table S3.2** for more details). We also noted transported and consumed fruits of *Parinari excelsa* and *Ficus sp.* below one feeding perch with bat guano (not included in the Table).

**Table S2**. Occurrence of fruiting tree species in foraging patches of the managed rainforest and agricultural lands, and occurrence of bat guano near individual fruiting trees.

| **Taxon name** | **Vegetation type** | **Occurrence of fruiting tree species in foraging patches** | | | **Occurrence of bat guano near individual trees** | **Fruiting phenology** | **Known to be consumed** |
| --- | --- | --- | --- | --- | --- | --- | --- |
|  |  | **Managed rainforest (N = 24)** | | **Agricultural lands (N = 37)** |  |  |  |
| Apocynaceae | Liana | | 4% (1) | - | - | - | - |
| *Chrysophyllum lacourtiana* | Tree | | 4% (1) | 3% (1) | - | - | Primates [1,2], *Potamochoerus porcus* [3] |
| *Cissus dinklagei* | Liana | | - | 8% (1) | 2/3 | Mdry, mdry [4,5] | Primates [6–8] |
| *Dacryodes edulis* | Cultivated tree | | - | 5% (1) | 1/2 | mdry [personal observation] | *Homo sapiens*, Primates [7,9] |
| *Desplatsia dewevrei* | Tree | | 4% (1) | - | - | Extended period, mainly Mwet and mwet [10] | *Gorilla Gorilla* [2] |
| *Elaeis guineensis* | Cultivated tree | | - | 3% (1) | - | All the year [4] | *H. sapiens*, Primates, domestic animals [11–13] |
| *Elaeis sp.* | Cultivated tree | | - | 3% (1) | - | - | *H. sapiens* |
| *Ficus mucuso* | Tree | | - | 8% (1) | 1/3 | All the year [4,14] | *Pan troglodytes* [11] |
| *Leplea thomsonnii* | Tree | | 4% (1) | - | 1/1 | - | *Ceratogymna spp.* [9] |
| *Lophira alata* | Tree | | - | 3% (1) | - | mdry [4] | - |
| *Macaranga barteri* | Tree | | 4% (1) | 5% (1 – 2) | 1/4 | Mdry, mdry [4] | *Cercopithecus mona* [8] |
| *Macaranga monandra* | Tree | | - | 3% (1) | 1/1 | Mdry [4] | - |
| *Macaranga sp.* | Tree | | - | 5% (1) | 1/2 | - | Bats [15] |
| *Microdesmis kasaiensis* | Tree | | 4% (1) | - | - | - | - |
| *Musanga cecropioides* | Tree | | 8% (1) | 41% (1 – 4) | 7/21 | All the year [11] | Primates [1,2,6,8], *Ceratogymna spp*. [9] |
| *Panda oleosa* | Tree | | 13% (1) | 3% (1) | - | Extended period, mainly Mwet and mdry [9] | *H. sapiens* [16], *C. mona* [8] |
| *Persea americana* | Cultivated tree | | - | 3%(2) | 2/2 | - | *H. sapiens* |
| *Raphia sp.* | Tree | | - | 3%(1) | - | - | - |
| *Strombosia grandifolia* | Tree | | 8%(1) | - | - | - | *C. mona* [8] |
| *Tiliacora sp.* | Tree | | 4% (1) | - | - | - | *G. gorilla* [2] |
| *Uapaca mole* | Tree | | 4% (1) | - | - | mwet [5] | *Ceratogymna spp.* [9], *Cercopithecus spp.* [1,8] |
| *Voacanga chalotiana* | Tree | | 4% (1) | 3% (1) | 1/2 | - |  |
| *Xylopia hypolampra* | Tree | | 4% (1) | - | - | All the year [4,5] | *Ceratogymna spp.* [9], *Cercopithecus spp.* [1] |
| Unidentified species | - | | 13% (1 – 2) | 3% (1) | 1/5 | - | - |

The ‘Occurrence of fruiting trees species in foraging patches‘ column indicates the percentage of foraging patches containing a given fruiting tree species per habitat type (the managed rainforest and agricultural lands). The number in bracket represents the range of the number of individual trees encountered in a single foraging patch. The ‘Occurrence of bat guano’ column indicates the number of times bat guano was present near an individual tree (number preceding the slash). The number following the slash represents the total number of tree prospected for guano. We also noted transported and consumed fruits of *Parinari excelsa* and *Ficus sp.* below one feeding perch with bat guano (not included in the Table)*.* Abbreviations: Mdry = major dry season; mdry = minor dry season; Mwet = major wet season; mwet = minor wet season.

**References**

^1^ Doran DM, McNeilage A, Greer D, Bocian C, Mehlman P, Shah N. 2002. Western lowland gorilla diet and resource availability: New evidence, cross-site comparisons, and reflections on indirect sampling methods. Am J Primatol. 58(3):91–116. doi:https://doi.org/10.1002/ajp.10053.

^2^ Gautier-Hion A. 1980. Seasonal Variations of Diet Related to Species and Sex in a Community of Cercopithecus Monkeys. J Anim Ecol. 49(1):237. doi:10.2307/4287.

^3^ Beaune D, Bollache L, Fruth B, Bretagnolle F. 2012. Bush pig ( Potamochoerus porcus ) seed predation of bush mango ( Irvingia gabonensis ) and other plant species in Democratic Republic of Congo. Afr J Ecol. 50(4):509–512. doi:10.1111/j.1365-2028.2012.01345.x.

^4^ Gautier-Hion A, Duplantier J-M, Emmons L, Feer F, Heckestweiler P, Moungazi A, Quris R, Sourd C. 1985. Coadaptation entre rythmes de fructification et frugivorie en forêt tropicale humide du Gabon : mythe ou réalité. Rev Ecol Terre Vie. 40(4):405–434.

^5^ White LJT. 1994. Patterns of fruit-fall phenology in the Lopé Reserve, Gabon. J Trop Ecol. 10(3):289–312. doi:10.1017/S0266467400007975.

^6^ Astaras C, Waltert M. 2010. What does seed handling by the drill tell us about the ecological services of terrestrial cercopithecines in African forests? Anim Conserv. 13(6):568–578. doi:https://doi.org/10.1111/j.1469-1795.2010.00378.x.

^7^ Hare B, Yamamoto S, editors. 2015. Bonobo cognition and behaviour. Leiden, The Netherlands ; Boston: Brill.

^8^ Sourd C, Gautier-Hion A. 1986. Fruit Selection by a Forest Guenon. J Anim Ecol. 55(1):235. doi:10.2307/4704.

^9^ Whitney KD, Fogiel MK, Lamperti AM, Holbrook KM, Stauffer DJ, Hardesty BD, Parker VT, Smith TB. 1998. Seed dispersal by Ceratogymna hornbills in the Dja Reserve, Cameroon. J Trop Ecol. 14(3):351–371. doi:10.1017/S0266467498000273.

^10^ Danquah, E., Oppong, S.K., 2007. Phenology of forest trees favoured by elephants in the Kakum Conservation Area, Ghana. Pachyderm 43–51.

^11^ Yamakoshi G. 1998. Dietary responses to fruit scarcity of wild chimpanzees at Bossou, Guinea: Possible implications for ecological importance of tool use. Am J Phys Anthropol. 106(3):283–295. doi:https://doi.org/10.1002/(SICI)1096-8644(199807)106:3<283::AID-AJPA2>3.0.CO;2-O.

^12^ Dahlan I, Islam M, Rajion MA. 2000. Nutrient Intake and Digestibility of Fresh, Ensiled and Pelleted Oil Palm (Elaeis guineensis) Frond by Goats. Asian-Australas J Anim Sci. 13(10):1407–1413. doi:10.5713/ajas.2000.1407.

^13^ Olaleru F, Onadeko AB, Ogunjemite BG, Egonmwan RI, Lambert JE. 2020. Diet and Nutritional Profile of the Mona Monkey (Cercopithecus mona, Schreber, 1774) in Okomu National Park, Nigeria: Preliminary Study. Afr Primates. 14:1–10.

^14^ Wakefield ML. 2008. Grouping Patterns and Competition Among Female Pan troglodytes schweinfurthii at Ngogo, Kibale National Park, Uganda. Int J Primatol. 29(4):907–929. doi:10.1007/s10764-008-9280-7.

^15^ Mohd-Azlan J, Tuen A, Abd Rahman M. 2010. Preliminary assessment of activity pattern and diet of the lesser dogfaced fruit bat Cynopterus brachyotis in a Dipterocarp Forest,Sarawak, Borneo. Trop Ecol. 51:175–180.

^16^ Fungo R, Muyonga J, Ngondi J, Mikolo-Yobo C, Iponga D, Ngoye A, Nchuaji Tang E, Chupezi Tieguhong J. 2019. Nutrient and Bioactive Composition of Five Gabonese Forest Fruits and Their Potential Contribution to Dietary Reference Intakes of Children Aged 1–3 Years and Women Aged 19–60 Years. Forests. 10(2):86. doi:10.3390/f10020086.

**Appendix S3**

**Methods**

We tested whether the closest distance between a foraging area (FA) used by a bat during the night and the river was influenced by the habitat type of the FA and the duration spent by the bat in the lek during that night. Several GLMMs (Gamma error distribution and log link function) were computed, including a null model and all effect combinations of these variables. A random intercept effect according to individuals was added. The random slope according to individuals was added for the lek duration only due to convergence issue. Akaike’s information criterion corrected for small sample sizes (AICc) was used to select the best modelon the basis of the ΔAICc and ωAICc (**Table S3.1**; see the main text for more details on the model selection procedure). Estimates (estimated marginal means) with their 95% confidence interval (CI) from the model retained are provided in **Table S3.2**. The best model includes the additive effect of the two candidate predictors (ΔAICc of the following model = 1.21, ωAICc = 0.52; following ωAICc = 0.29; Rm² = 0.09, Rc² = 0.24; **Table S3.1**). As a result, foraging areas used in managed rainforest by a bat during the night were on average located nearest to the river compared to FAs located in agricultural lands (**Figure S3a**). In addition, the mean distance between FAs used by that bat during the night and the river tend to decrease with the time spent by the bat in the lek during that night (**Figure S3b**).

**Table S3.1.** Model selection result for the variation on the mean distance between a foraging area (FA) used by a bat during the night and the river.

| Response variable | Fixed effects | Error distribution | Link function | AICc | ΔAICc | ωAICc |
| --- | --- | --- | --- | --- | --- | --- |
|  |  |  |  |  |  |  |
| **FA-river distance** | **Habitat + Lek duration** | Gamma | log | **7606.83** | **0.00** | **0.52** |
|  | Habitat | Gamma | log | 7608.04 | 1.21 | 0.29 |
|  | Habitat *Lek duration | Gamma | log | 7608.87 | 2.04 | 0.19 |
|  | Lek duration | Gamma | log | 7645.19 | 38.36 | 0.00 |
|  | Null model | Gamma | log | 7648.81 | 41.99 | 0.00 |

FA-river distance: the closest distance between a given FA used by a bat during the night and the river. Habitat: two habitat types (i.e. agricultural lands and managed rainforest) associated to a FA. Lek duration: the total duration spent in the lek by a bat during the night. Null model: intercept-only model. AICc: the Akaike's information criterion corrected for small sample sizes. ΔAICc: the difference in AICc between any model and the model with the lowest AICc. ωAICc: may be considered as the probability that a given model is the best approximation (Akaike weight). The model retained on the basis of these criteria is shown in bold.

**Table S3.2.** Estimates with their 95% confidence interval (CI) resulting from the model (GLMM with Gamma error distribution and log link function) retained for the variation on the mean distance between a foraging area (FA) used by a bat during the night and the river.

| Response variable | Parameter | Mean estimate | CI (95%) |
| --- | --- | --- | --- |
|  |  |  |  |
| FA-river distance | Intercept | 6.34 | 6.11;6.58 |
|  | Habitat | 0.57 | 0.40;0.76 |
|  | Lek duration | -0.10 | -0.27;0.06 |

FA-river distance: the closest distance between a given FA used by a bat during the night and the river. Habitat: parameter associated to the type ‘agricultural lands’ (‘the managed rainforest’ being the implicit reference level in the intercept component). Lek duration: the total duration spent in the lek by a bat during the night.


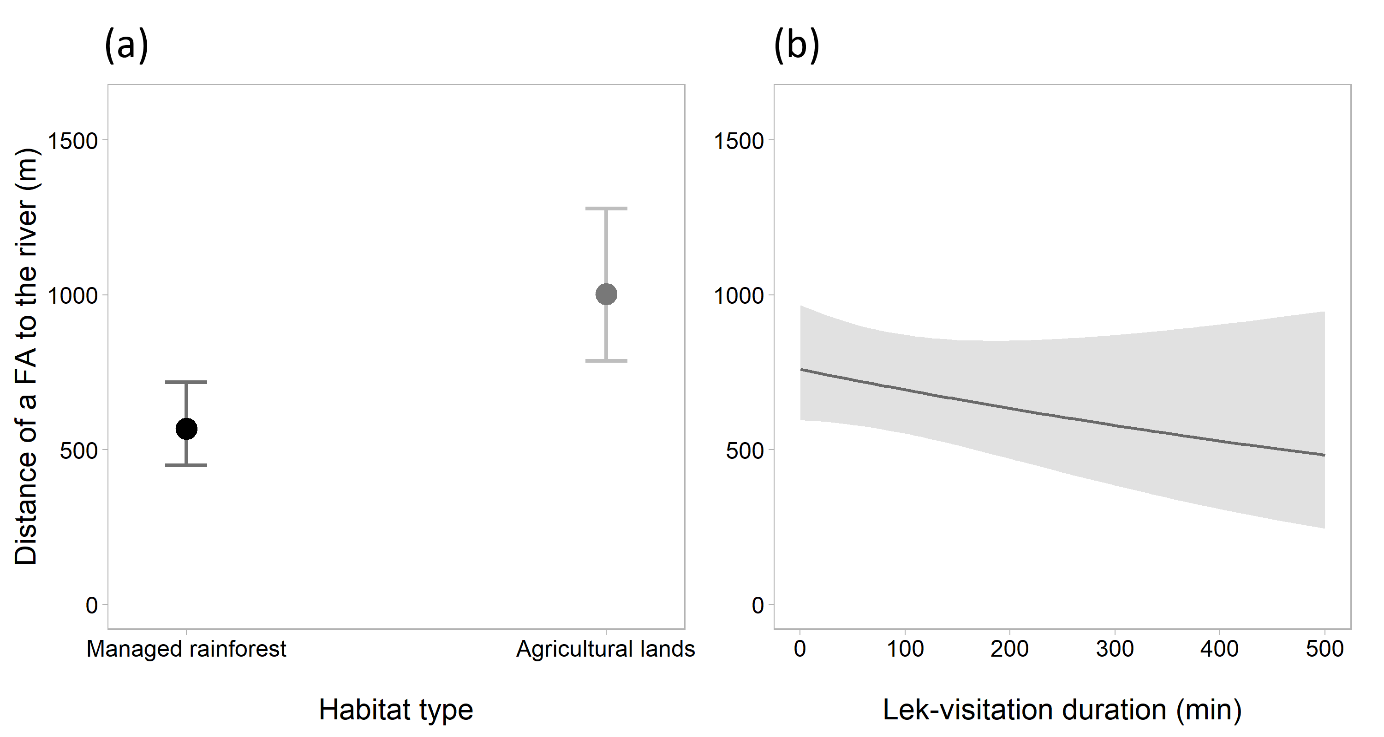


**Figure S3.** Estimation (with 95% CI; n = 16 individuals) of the distance of a foraging area (FA) selected to the river (in m), according to (a) the habitat type of the FA, and (b) the total duration spent in the lek by the bat during the night (in min).

**Appendix S4**

**Table S4.** Summary of individual features, then mating and foraging behaviours of the 28 H. monstrosus equipped with GPS loggers.

| Bat ID | Weight (g) | Group | Number of nights | Probability of visiting the lek | Mean distance between the roost and the lek (m) | Mean visitation duration of the lek (min) | Number of GPS foraging locations in the managed rainforest | Number of GPS foraging locations in agricultural lands | Number of FAs visited during the study period | Mean number of FAs visited in the managed rainforest | Mean number of FAs visited in agricultural lands | Mean visitation duration of a FA in the managed rainforest (min) | Mean visitation duration of a FA in agricultural lands (min) |
| --- | --- | --- | --- | --- | --- | --- | --- | --- | --- | --- | --- | --- | --- |
| bat7240 | 327 | 1 | 11 | 0.00 | 8763 ± 17 | - | 815 | 262 | 24 | 5.2 ± 1.9 | 1.1 ± 0.7 | 71 ± 70 | 112 ± 93 |
| bat7243 | 416 | 1 | 11 | 1.00 | 1779 ± 41 | 131 ± 57 | 493 | 648 | 4 | 1.5 ± 0.5 | 0.9 ± 0.3 | 154 ± 113 | 324 ± 147 |
| bat7250 | 421 | 1 | 10 | 1.00 | 1031 ± 16 | 240 ± 89 | 101 | 599 | 7 | 1.0 ± 0.9 | 1.0 ± 0.0 | 50 ± 47 | 300 ± 80 |
| bat7253 | 394 | 1 | 12 | 0.42 | 1341 ± 995 | 278 ± 145 | 23 | 1233 | 6 | 0.2 ± 0.9 | 1.5 ± 0.7 | 33 ± 16 | 343 ± 250 |
| bat7254b | 435 | 1 | 10 | 0.20 | 5317 ± 2180 | 445 ± 78 | 442 | 630 | 13 | 2.6 ± 2 | 1.5 ± 0.5 | 85 ± 70 | 210 ± 150 |
| bat7440 | 423 | 1 | 6 | 0.00 | 1839 ± 26 | - | 0 | 811 | 2 | 0.0 | 2.0 ± 0.0 | - | 338 ± 310 |
| bat7446 | 315 | 1 | 10 | 0.80 | 3780 ± 5075 | 285 ± 128 | 219 | 503 | 10 | 1.2 ± 1.8 | 1.3 ± 0.9 | 91 ± 48 | 193 ± 84 |
| bat7447 | 305 | 1 | 10 | 0.00 | 1339 ± 21 | - | 59 | 1160 | 4 | 0.3 ± 0.7 | 1 ± 0.0 | 98 ± 49 | 580 ± 86 |
| bat7455b | 405 | 1 | 11 | 0.09 | 2362 ± 2484 | 70 | 202 | 1300 | 6 | 0.9 ± 1.4 | 1 ± 0.0 | 101 ± 71 | 591 ± 197 |
| bat7463 | 370 | 1 | 11 | 0.00 | 4488 ± 48 | - | 626 | 762 | 6 | 1.9 ± 0.7 | 0.9 ± 0.3 | 149 ± 130 | 381 ± 118 |
| bat7464 | 428 | 1 | 11 | 1.00 | 5183 ± 1766 | 130 ± 83 | 314 | 519 | 10 | 2.5 ± 1.3 | 2.5 ± 0.5 | 57 ± 37 | 93 ± 69 |
| bat7467 | 397 | 1 | 12 | 0.00 | 8682 ± 42 | - | 652 | 502 | 11 | 3.2 ± 0.7 | 1.9 ± 0.9 | 86 ± 89 | 109 ± 110 |
| bat7470 | 392 | 1 | 10 | 0.10 | 3755 ± 4893 | 15 | 1074 | 235 | 6 | 2.0 ± 1.3 | 0.0 | 327 ± 276 | - |
| bat7472 | 432 | 1 | 5 | 1.00 | 793 ± 11 | 275 ± 25 | 223 | 60 | 7 | 4.2 ± 0.8 | 1 ± 0 | 53 ± 37 | 62 ± 9 |
| bat7473 | 416 | 1 | 9 | 0.89 | 1969 ± 4680 | 330 ± 111 | 220 | 374 | 10 | 1.7 ± 2.1 | 1 ± 0 | 73 ± 67 | 208 ± 79 |
| bat7483 | 392 | 1 | 8 | 0.62 | 4476 ± 36 | 61 ± 32 | 454 | 391 | 10 | 2.8 ± 0.7 | 1.4 ± 0.7 | 105 ± 105 | 175 ± 143 |
| bat7242 | 411 | 2 | 18 | 0.06 | 2375 ± 1173 | - | 90 | 410 | - | - | - | - | - |
| bat7244 | 439 | 2 | 3 | 1.00 | 7635 ± 5368 | - | 87 | 3 | - | - | - | - | - |
| bat7247 | 455 | 2 | 20 | 0.75 | 5700 ± 2334 | - | 412 | 191 | - | - | - | - | - |
| bat7249 | 447 | 2 | 3 | 1.00 | 886 ± 684 | - | 2 | 70 | - | - | - | - | - |
| bat7251 | 416 | 2 | 21 | 0.38 | 6068 ± 2984 | - | 217 | 357 | - | - | - | - | - |
| bat7252 | 420 | 2 | 17 | 0.35 | 4361 ± 3135 | - | 194 | 446 | - | - | - | - | - |
| bat7439 | 408 | 2 | 3 | 0.67 | 835 ± 18 | - | 0 | 69 | - | - | - | - | - |
| bat7450 | 427 | 2 | 18 | 0.28 | 3750 ± 1253 | - | 287 | 337 | - | - | - | - | - |
| bat7457 | 335 | 2 | 19 | 0.26 | 6282 ± 4561 | - | 345 | 418 | - | - | - | - | - |
| bat7475 | 287 | 2 | 19 | 0.00 | 11781 ± 1123 | - | 443 | 296 | - | - | - | - | - |
| bat7481 | 431 | 2 | 13 | 0.92 | 2975 ± 2893 | - | 204 | 146 | - | - | - | - | - |
| bat7482 | 423 | 2 | 16 | 0.00 | 759 ± 26 | - | 42 | 317 | - | - | - | - | - |

Weight is the mass of bats during the catching event. The number in the ‘Group’ column referred to the schedule used for GPS data acquisition (group 1: one location recorded every five minutes; group 2: one location recorded every five minutes during high activity bouts and one location every 30 minutes during low activity bouts; see the Methods of the main text for further details). Number of nights is the total number of nights during which the GPS was active. Number of GPS foraging locations and total number of FAs (foraging areas) visited are given in cumulated values during the period in which the GPS was active. Probability of visiting the lek, mean visitation duration of the lek, mean distance between the roost and the lek, mean number and mean visitation duration of FAs represent the overall behaviour of a bat a given night. Standard deviations were given for each mean value. All individuals of *Hypsignathus monstrosus* represented in the table are adult mature males.


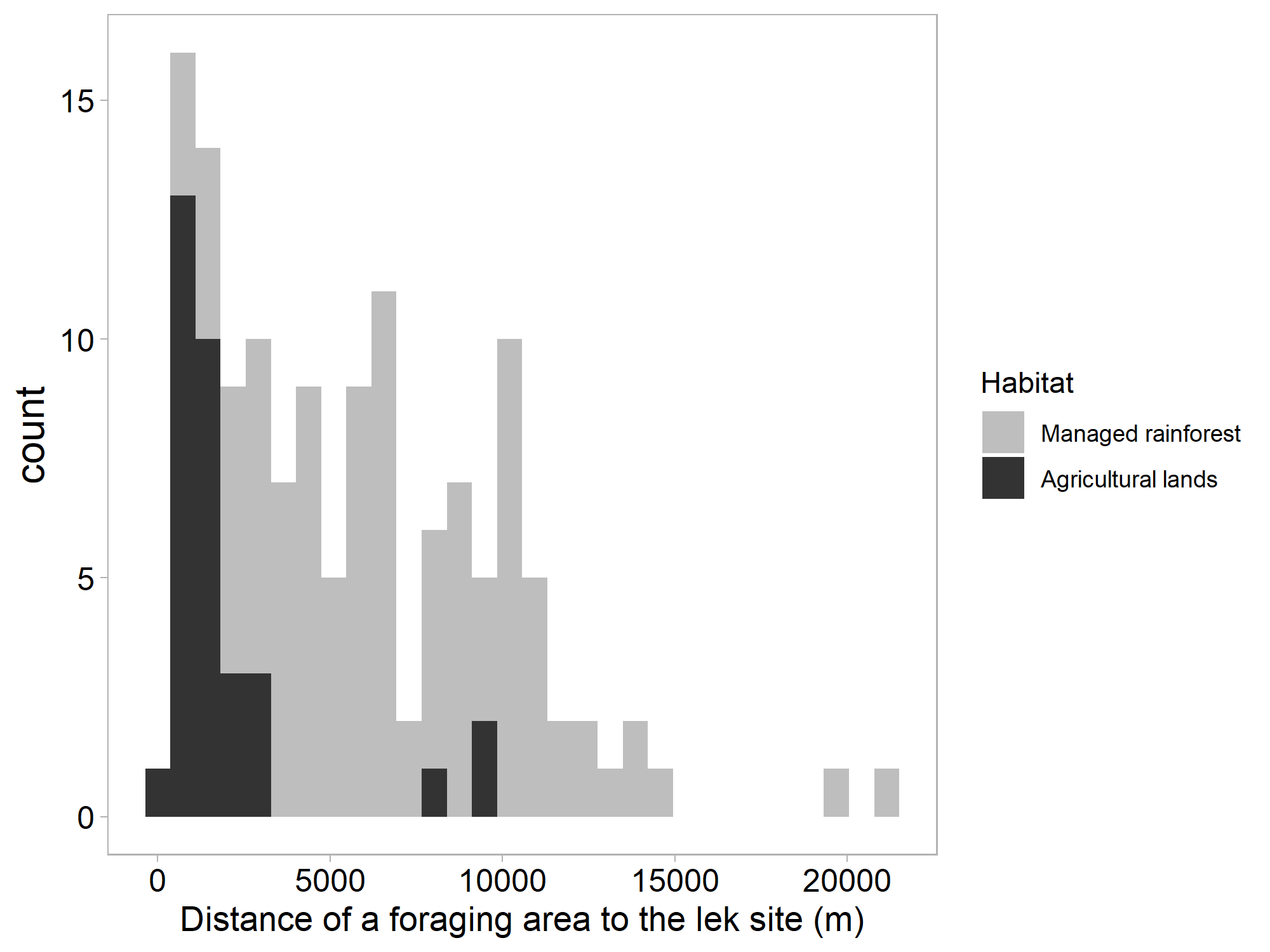


**Figure S4.1**. Distribution of the distance of a foraging area to the lek site (in m), according to the habitat type. The distribution includes 136 foraging areas from 16 individuals of *Hypsignathus monstrosus* (group 1). The distance considered is the closest distance. Details on foraging area characterization are provided in the Methods.

**
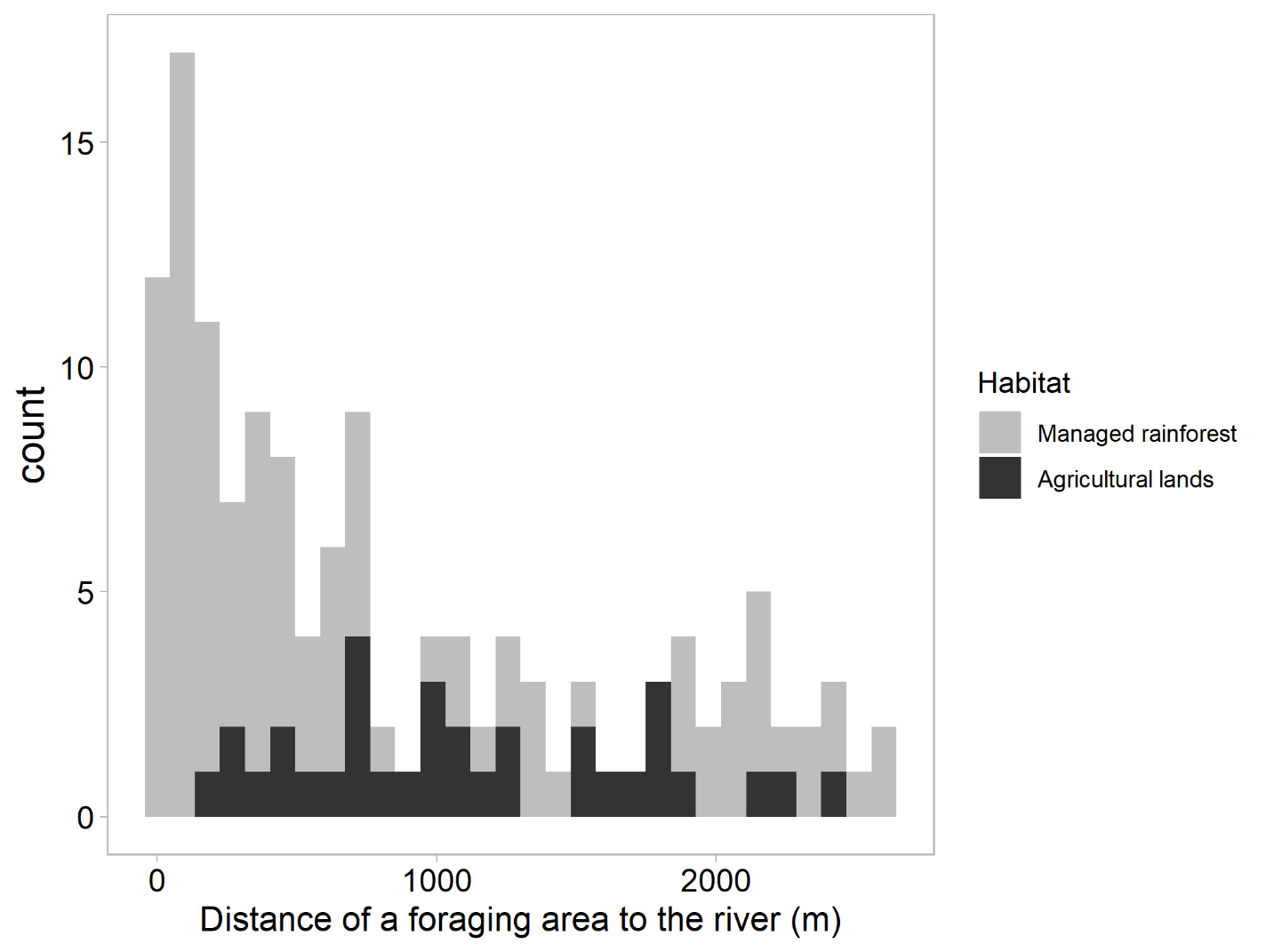
**

**Figure S4.2**. Distribution of the distance of a foraging area to the river (in m), according to the habitat type. The distribution includes 136 foraging areas from 16 individuals of *Hypsignathus monstrosus* (group 1). Details on foraging area characterization are provided in Methods.

**Appendix S5**

**Table S5.** Model selection results for foraging habitat selection pattern of the population studied, and for foraging activity pattern of individuals during the night.

| Pattern | Response variable | Fixed effects | Error distribution | Link function | AICc | ΔAICc | ωAICc |
| --- | --- | --- | --- | --- | --- | --- | --- |
|  |  |  |  |  |  |  |  |
| RSF |  |  |  |  |  |  |  |
|  | **Probability of use** | **Habitat * Location-river distance** | binomial | logit | **386882.1** | **0.00** | **1.00** |
|  |  | Habitat + Location-river distance | binomial | logit | 387280.8 | 398.72 | 0.00 |
|  |  | Habitat | binomial | logit | 398240.8 | 11358.73 | 0.00 |
|  |  | Location-river distance | binomial | logit | 428057.1 | 41175.05 | 0.00 |
|  |  | Null model | binomial | logit | 428063.5 | 41181.39 | 0.00 |
|  |  |  |  |  |  |  |  |
| Foraging activity |  |  |  |  |  |  |  |
|  | **FA-lek distance** | **Habitat * Lek duration** | Gamma | log | **8802.18** | **0.00** | **1.00** |
|  |  | Habitat + Lek duration | Gamma | log | 8815.72 | 13.54 | 0.00 |
|  |  | Habitat | Gamma | identity | 8826.86 | 24.68 | 0.00 |
|  |  | Lek duration | Gamma | log | 9245.26 | 443.08 | 0.00 |
|  |  | Null model | Gamma | log | 9267.56 | 465.38 | 0.00 |
|  |  |  |  |  |  |  |  |
|  |  |  |  |  |  |  |  |
|  | **Number of FA** | **Habitat + Habitat:Lek duration** | poisson | log | **924.70** | **0.00** | **1.00** |
|  |  | Habitat | poisson | log | 949.30 | 24.61 | 0.00 |
|  |  | Null model | poisson | log | 969.14 | 44.45 | 0.00 |
|  |  |  |  |  |  |  |  |
|  |  |  |  |  |  |  |  |
|  | **FA duration** | **Habitat * FA-river distance + Lek duration** | Gamma | log | **5839.12** | **0.00** | **0.72** |
|  |  | Habitat + FA-river distance + Lek duration | Gamma | log | 5844.11 | 4.99 | 0.06 |
|  |  | Habitat + Lek duration | Gamma | log | 5844.13 | 5.00 | 0.06 |
|  |  | Habitat * Lek duration | Gamma | log | 5844.26 | 5.13 | 0.06 |
|  |  | Habitat * Lek duration + FA-river distance | Gamma | log | 5844.45 | 5.33 | 0.05 |
|  |  | Habitat * FA-river distance | Gamma | log | 5845.15 | 6.02 | 0.04 |
|  |  | Habitat + FA-river distance | Gamma | log | 5847.87 | 8.75 | 0.01 |
|  |  | Habitat | Gamma | log | 5848.75 | 9.63 | 0.01 |
|  |  | FA-river distance | Gamma | log | 5913.41 | 74.29 | 0.00 |
|  |  | FA-river distance + Lek duration | Gamma | log | 5915.45 | 76.33 | 0.00 |
|  |  | Null model | Gamma | log | 5925.06 | 85.93 | 0.00 |
|  |  | Lek duration | Gamma | log | 5927.08 | 87.96 | 0.00 |
|  |  |  |  |  |  |  |  |
|  |  |  |  |  |  |  |  |
|  | **FA-revisitation probability** | **FA duration** | binomial | logit | **316.13** | **0.00** | **0.48** |
|  |  | FA duration + Habitat | binomial | logit | 318.07 | 1.94 | 0.18 |
|  |  | FA duration + FA-river distance | binomial | logit | 318.14 | 2.01 | 0.18 |
|  |  | Habitat * FA-river distance + FA duration | binomial | logit | 319.65 | 3.52 | 0.08 |
|  |  | Habitat + FA-river distance + FA duration | binomial | logit | 319.88 | 3.75 | 0.07 |
|  |  | Habitat * FA duration | binomial | logit | 320.00 | 3.87 | 0.06 |
|  |  | Habitat * FA duration + FA-river distance | binomial | logit | 321.83 | 5.70 | 0.03 |
|  |  | Habitat | binomial | logit | 361.89 | 45.76 | 0.00 |
|  |  | Habitat * FA-river distance | binomial | logit | 364.84 | 48.71 | 0.00 |
|  |  | Habitat + FA-river distance | binomial | logit | 366.31 | 50.18 | 0.00 |
|  |  | FA-river distance | binomial | logit | 371.40 | 55.27 | 0.00 |
|  |  | Null model | binomial | logit | 374.50 | 58.37 | 0.00 |

Habitat: two habitat types (i.e. agricultural lands and the managed rainforest) associated to a specific location. Location-river distance: the closest distance between a given location and the river. Lek duration: the total duration spent in the lek by a bat during the night. FA-river distance: the closest distance between a given foraging area (FA) and the river. FA duration: the total duration spent in a given FA by a bat during the night. Null model: intercept-only model. Each model was a GLMM (computed with ‘lme4’ R package) for which the error-distribution family and link function are specified. A random intercept effect according to individuals was added in all models. The random slope according to individuals was added for each predictor only if no convergence issues occurred due to this effect for any other model belonging to the same analysis – as follows: the location-river distance for the ‘Probability of use’ (RSF) model, the roost-lek distance for the ‘lek-visitation probability’ model, and the lek duration for the ‘number of FAs’ model. Quantitative explanatory variables were centred and scaled. AICc: the Akaike's information criterion corrected for small sample sizes. ΔAICc: the difference in AICc between any model and the model with the lowest AICc. ωAICc: may be considered as the probability that a given model is the best approximation (Akaike weight). The model retained is shown in bold for each response variable (additional details are given in the main text).


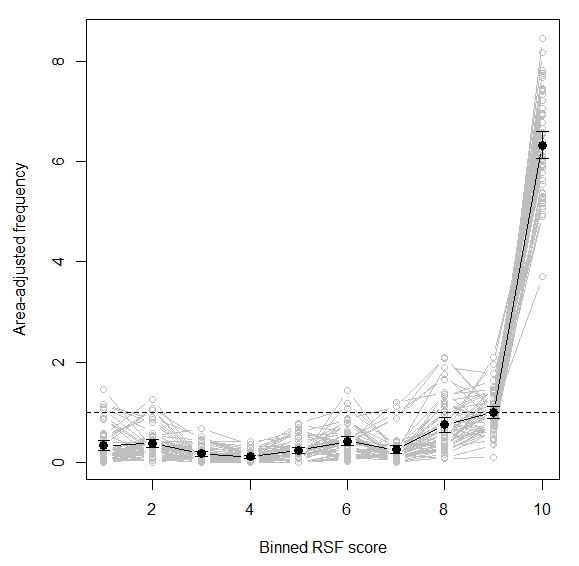


**Figure S5**. RSF model validation, following (Boyce et al. 2002). Lines with open circles correspond to each iteration (10 iterations). The line with black dots shows the mean of these iterations. An area-adjusted frequency of 1 would indicate that cross-validated use locations occurred at rates expected by chance.

**Reference**

Boyce MS, Vernier PR, Nielsen SE, Schmiegelow FKA. 2002. Evaluating resource selection functions. Ecol Model. 157(2002):281–300. doi:10.1016/S0304-3800(02)00200-4.
